# Stomatal optimization based on xylem hydraulics (SOX) improves land surface model simulation of vegetation responses to climate

**DOI:** 10.1111/nph.16419

**Published:** 2020-02-17

**Authors:** Cleiton B. Eller, Lucy Rowland, Maurizio Mencuccini, Teresa Rosas, Karina Williams, Anna Harper, Belinda E. Medlyn, Yael Wagner, Tamir Klein, Grazielle S. Teodoro, Rafael S. Oliveira, Ilaine S. Matos, Bruno H. P. Rosado, Kathrin Fuchs, Georg Wohlfahrt, Leonardo Montagnani, Patrick Meir, Stephen Sitch, Peter M. Cox

**Affiliations:** ^1^ College of Life and Environmental Sciences University of Exeter Exeter EX4 4QF UK; ^2^ Department of Plant Biology University of Campinas Campinas 13083‐862 Brazil; ^3^ CREAF Bellaterra 08193 Barcelona Spain; ^4^ ICREA Pg. Lluís Companys 23 08010 Barcelona Spain; ^5^ Met Office Hadley Centre Fitzroy Road Exeter EX1 3PB UK; ^6^ College of Engineering, Mathematics and Physical Sciences University of Exeter Exeter EX4 4QF UK; ^7^ Hawkesbury Institute for the Environment Western Sydney University Locked Bag 1797 Penrith NSW 2751 Australia; ^8^ Department of Plant & Environmental Sciences Weizmann Institute of Science 76100 Rehovot Israel; ^9^ Institute of Biological Sciences Federal University of Pará Belém 66075‐110 Brazil; ^10^ Department of Ecology – IBRAG Rio de Janeiro State University (UERJ) Rio de Janeiro 20550‐013 Brazil; ^11^ Department of Environmental Systems Science ETH Zurich Universitätstrasse 2 8092 Zurich Switzerland; ^12^ Department of Ecology University of Innsbruck Innsbruck 6020 Austria; ^13^ Forest Services Autonomous Province of Bolzano Via Brennero 6 39100 Bolzano Italy; ^14^ Research School of Biology The Australian National University Acton ACT 2601 Australia; ^15^ School of Geosciences University of Edinburgh Edinburgh EH9 3FF UK

**Keywords:** climate change, drought, eddy covariance, land‐surface models, stomatal optimization, xylem hydraulics

## Abstract

- Land surface models (LSMs) typically use empirical functions to represent vegetation responses to soil drought. These functions largely neglect recent advances in plant ecophysiology that link xylem hydraulic functioning with stomatal responses to climate.
- We developed an analytical stomatal optimization model based on xylem hydraulics (SOX) to predict plant responses to drought. Coupling SOX to the Joint UK Land Environment Simulator (JULES) LSM, we conducted a global evaluation of SOX against leaf‐ and ecosystem‐level observations.
- SOX simulates leaf stomatal conductance responses to climate for woody plants more accurately and parsimoniously than the existing JULES stomatal conductance model. An ecosystem‐level evaluation at 70 eddy flux sites shows that SOX decreases the sensitivity of gross primary productivity (GPP) to soil moisture, which improves the model agreement with observations and increases the predicted annual GPP by 30% in relation to JULES. SOX decreases JULES root‐mean‐square error in GPP by up to 45% in evergreen tropical forests, and can simulate realistic patterns of canopy water potential and soil water dynamics at the studied sites.
- SOX provides a parsimonious way to incorporate recent advances in plant hydraulics and optimality theory into LSMs, and an alternative to empirical stress factors.

## Abstract 2

See also the Commentary on this article by Anderegg & Venturas, **226**: 1535–1538.

## Introduction

Large areas of the globe will be exposed to increased aridity in the near future (Sheffield & Wood, 2008; Duffy *et al*., 2015; Marengo *et al*., 2018). As drought events become more intense and frequent, accurately representing vegetation–climate feedbacks in Earth system models (ESMs) is increasingly important, as these interactions can drastically influence model projections of global climate change (Cox *et al*., 2000). The current generation of land surface models (LSMs) does not accurately simulate vegetation carbon dynamics during drought (Sitch *et al*., 2008; Powell *et al*., 2013; Medlyn *et al*., 2016; Ukkola *et al*., 2016; Restrepo‐Coupe *et al*., 2017; Rogers *et al*., 2017; Eller *et al*., 2018b), thereby restricting our capability to predict the effect of increased aridity on vegetation distribution and its feedbacks on the global carbon cycle and climate. Many LSMs represent the effects of reduced soil moisture on canopy carbon assimilation (*A*) using an empirical drought factor commonly referred as *β*‐factor (Cox *et al*., 1998). The *β*‐factor approach has been shown to overestimate plant responses to seasonal and experimentally induced drought (Ukkola *et al*., 2016; Restrepo‐Coupe *et al*., 2017; Eller *et al*., 2018b). The *β*‐factor has a large impact on the modelled global carbon budget, supressing 30–40% of the annual gross primary productivity (GPP) in large areas of arid and semiarid ecosystems (Trugman *et al.,* 2018). Despite its importance, there is scarce empirical support for the drought functions used in most LSMs (Medlyn *et al*., 2016). The lack of a theoretical or empirical basis for the *β*‐factor implies an urgent need for new modelling approaches to replace this important component of LSMs so as to improve our capacity to predict vegetation–climate interactions.

Stomatal responses of plants to soil drought involve complex chemical signalling and hydrodynamic processes in leaf cells, some of which have not been entirely elucidated (Buckley, 2017, 2019; Qu *et al*., 2019). Stomatal optimization models are a useful approach to model stomatal behaviour that circumvents the need to explicitly represent the physiological processes involved in stomatal regulation. Optimization models employ a ‘goal‐oriented’ approach, assuming that plant stomata behaviour has been selected through plant evolutionary history to maximize a given objective function (Cowan, 2002; Dewar *et al*., 2009; Prentice *et al*., 2014; Buckley, 2017). The traditional approach to model optimal stomatal behaviour is derived from the seminal work of Cowan & Farquhar (1977). This approach proposes that optimal stomatal behaviour maximizes *A* minus the carbon cost of water lost (*λE*) over a given time interval, where *E* is transpiration and *λ* is the Lagrange multiplier that represents the carbon cost of a unit of water lost. This model, hereafter labelled CF, after Cowan and Farquhar, is capable of simulating many patterns of stomatal responses to climate over short timescales (Farquhar *et al*., 1980; Berninger & Hari, 1993), and has provided the theoretical basis for several widely used semi‐empirical stomatal models (Jacobs, 1994; Leuning, 1995; Medlyn *et al*., 2011). However, CF predicts that stomatal conductance (*g*_s_) increases in response to elevated CO_2_ when *A* is Rubisco‐limited, which contradicts most observations (Mott, 1988; Medlyn *et al*., 2001). Other limitations are related to the *λ*, as the CF hypothesis does not link *λ* to measurable plant traits or environmental quantities (Buckley, 2017), and assumes *λ* is constant over the period of reference (Cowan & Farquhar, 1977), which makes the original CF unable to predict long‐term *g*_s_ decline in response to soil moisture depletion.

Since the original CF work, many attempts have been made to incorporate the effects of declining soil moisture in the CF stomatal optimization framework (Cowan, 1986; Mäkelä *et al*., 1996; Williams *et al*., 1996; Manzoni *et al*., 2013). Some of these attempts, such as the soil–plant–atmosphere (SPA) model of Williams *et al*. (1996), employ principles of plant hydraulics to constrain stomatal optimization and have been successfully incorporated into LSMs (Bonan *et al*., 2014). The numerical approach used by SPA employs a hydraulic threshold to set a lower water potential limit (Ψ_min_) for *g*_s_, which simulates a strict isohydric stomatal regulation (Fisher *et al*., 2006). Despite using plant hydraulics, SPA still relies on a water‐use efficiency optimization similar to CF to model stomatal behaviour when Ψ > Ψ_min_ (Williams *et al*., 1996; Bonan *et al*., 2014).

Alternative routes to model plant optimal stomatal behaviour have been proposed recently (for a review, see Mencuccini *et al*., 2019a). These approaches circumvent the CF limitations by assuming that plant optimal stomatal behaviour minimizes the instantaneous fitness costs associated with low Ψ. These new optimization models use widely available plant hydraulic traits (Kattge *et al*., 2011; Choat *et al*., 2012) to simulate *g*_s_ responses to environmental conditions, producing a realistic *g*_s_ decline in response to elevated atmospheric CO_2_ and soil drought (Sperry *et al*., 2017; Venturas *et al*., 2018; Eller *et al*., 2018b; Wang *et al*., 2019). This approach predicts a tight coordination between stomatal and xylem functioning which is widely corroborated by observations (Hubbard *et al*., 2001; Meinzer *et al*., 2009; Klein, 2014). Another advantage of this approach is its capacity to simulate a diversity of contrasting stomatal behaviours, from iso‐ to anisohydric (Martinez‐Vilalta *et al*., 2014; Klein, 2014).

Sperry *et al*. (2017) proposes a model that assumes that, as xylem hydraulic conductance declines, the increased risk of hydraulic failure is the main fitness cost associated with low Ψ. Eller *et al*. (2018b) adapted the Sperry *et al*. (2017) model into the stomatal optimization model based on xylem hydraulics (SOX), which differs from the Sperry *et al*. (2017) model principally by using a different optimization target. The SOX optimization target is based on the PGEN model (Friend, 1995), which assumes that stomata optimize plant dry matter production, represented by the product of photosynthesis and a linear function of Ψ. The SOX model in Eller *et al*. (2018b) uses a numerical routine to find the optimum *g*_s_. However, the PGEN optimization target can also be found analytically (Friend & Cox, 1995; Dewar *et al*., 2018). A parsimonious analytical formulation for SOX would facilitate its incorporation into existing LSMs and provide a practical alternative to the *β*‐function for modelling stomatal responses to drought at global scales.

In this study we develop an analytical approximation for the numerical SOX model presented in Eller *et al*. (2018b). We then create a new configuration for the Joint UK Land Environment Simulator (JULES; Best *et al*., 2011; Clark *et al*., 2011) that uses SOX to compute vegetation *g*_s_ from environmental and plant hydraulic data. Using a global dataset of xylem hydraulic traits, together with an extensive leaf gas‐exchange and eddy covariance dataset, we calibrate the SOX parameters and compare the JULES‐SOX performance to the default JULES using the *β*‐function, across all major global biomes. Our goals in this paper are twofold: to test SOX agreement with global observations of *g*_s_ to assess the generality of the underlying hypothesis in SOX, that is, that plant stomata evolved to balance carbon assimilation with the loss of hydraulic conductance; and to evaluate the effect of SOX on JULES ecosystem‐scale predictions of carbon and water fluxes, and their agreement with observations.

## Materials and Methods

### Analytical SOX description

The SOX central hypothesis can be summarized as ‘stomatal conductance (*g*_s_) is such as to maximize the product of leaf photosynthesis and xylem hydraulic conductance’ and is given by:

(Eqn 1) $$A\left[c_{i}\left(g_{s}\right)\right]K\left[\Psi_{m}\left(g_{s}\right)\right],$$

where *A* is leaf net CO_2_ assimilation (mol CO_2_ m^−2^ s^−1^), which is a function of leaf internal CO_2_ partial pressure (*c*_i_, in Pa), which is itself a function of stomatal conductance to CO_2_ (*g*_s_, mol m^−2^ s^−1^). The *K* is the normalized (0–1) xylem hydraulic conductance computed as:

(Eqn 2) $$K\left(\Psi \right)=\frac{1}{\left[1+{\left(\Psi /\Psi_{50}\right)}^{a}\right]},$$

where Ψ_50_ is Ψ when *K *= 0.5 and the parameter *a* gives the shape of the curve, with a higher *a* producing a steeper response to Ψ. We use the mean (Ψ_m_, MPa) of the canopy water potential at the predawn (Ψ_pd_, MPa) and the canopy water potential (Ψ_c_, MPa) to compute *K* with Eqn 2 to account for the gradual decline in Ψ along the soil to canopy hydraulic pathway (see details in Supporting Information Notes S1). The *g*_s_ value that maximizes Eqn 1 is found at:

(Eqn 3) $$\frac{\partial AK}{\partial g_{s}}=0.$$

The *g*_s_ value that satisfies Eqn 3 was found numerically in Eller *et al*. (2018b), but a computationally efficient analytical solution is preferable for application in dynamic global vegetation models (DGVMs) and ESMs. We developed an analytical approximation for the optimal SOX *g*_s_ using the partial derivatives of *A* with respect to *c*_i_ and *K* with respect to Ψ_m_. All steps of the model derivation are described in Notes S1. The resulting SOX equation for the optimal *g*_s_ is:

(Eqn 4) $$g_{s}=0.5\frac{\partial A}{\partial c_{i}}\left(\sqrt{\frac{4\xi }{\partial A/\partial c_{i}}+1}-1\right),$$

The benefit of stomatal opening is represented here by the sensitivity of leaf photosynthesis to the internal CO_2_ concentration ($\partial A/\partial c_{i}$). By contrast, the parameter *ξ* represents the cost of stomatal opening in terms of loss of xylem conductivity under low Ψ_pd_ and/or higher leaf‐to‐air vapour pressure (*D*, mol mol^−1^):

(Eqn 5) $$\xi =\frac{2}{1/K\partial K/\partial \Psi_{m}r_{p}1.6D}.$$

Low *ξ* indicates high hydraulic costs occurring during drought (i.e. lower Ψ_pd_ and higher *D*; Fig. S1). SOX simulates dynamic changes on the plant hydraulic resistance (*r*_p_), computing *r*_p_ as a function of Ψ_pd_ and the plant minimum hydraulic resistance (*r*_pmin_, m^2^ s MPa mol^−1^ H_2_O):

(Eqn 6) $$r_{p}=\frac{r_{p,\min}}{K\left(\Psi_{\text{pd}}\right)}.$$

Solving SOX main equations (Eqns 4, 5) requires computing the partial derivatives of *A* and *K*, ∂*A*/∂*c*_i_ and ∂*K*/∂Ψ_m_, respectively. These derivatives were estimated numerically in this study as described in Notes S2.

We evaluated SOX as a stand‐alone leaf‐level model, and coupled to JULES (hereafter JULES‐SOX). The leaf‐level model was evaluated against leaf gas exchange data as an ‘assumption centred’ (*sensu* Medlyn *et al*., 2015) test of the hypothesis underlying SOX. The JULES‐SOX was then evaluated against ecosystem‐level eddy flux data, which constituted the first practical test of the utility of SOX for LSMs.

### JULES *β*‐function description

The JULES model (Best *et al*., 2011; Clark *et al*., 2011) uses the Collatz *et al*. (1991, 1992) photosynthesis model for C_3_ and C_4_ plants (Notes S3) to produce unstressed rates of *A* based on the colimitation of light, Rubisco carboxylation capacity, and the transport of photoassimilates (for C_3_ plants) and PEPcarboxylase limitation (for C_4_ plants). The effect of soil moisture in *A* in the default JULES is given by multiplying *A* by the *β* factor, computed using the *β*‐function from Cox *et al*. (1998):

(Eqn 7) $$\beta =\left\{\begin{array}{ccc} 1 & \text{for} & \theta >\theta_{c}\\ \frac{\theta -\theta_{w}}{\theta_{c}-\theta_{w}} & \text{for} & \theta_{w}<\theta \leq \theta_{c}\\ 0 & \text{for} & \theta \leq \theta_{w} \end{array},\right.$$

where *θ* is the mean soil moisture in the root zone (m^3^ m^−3^), and *θ*_c_ and *θ*_w_, are the critical and wilting points, which are defined by Cox *et al*. (1998) as the *θ* when soil Ψ is − 0.033 and − 1.5 MPa, respectively. The default JULES formulation employs the Jacobs (1994) equation to predict *c*_i_ from *D*,* c*_a_ and the CO_2_ compensation point, *Γ* (Pa):

(Eqn 8) $$c_{i}=f_{0}\left(1-\frac{D}{D_{\text{crit}}}\right)\left(c_{a}-\Gamma \right),$$

where *f*_0_ and *D*_crit_ are empirical parameters (Jacobs, 1994; Cox *et al*., 1998).

The JULES‐SOX configuration replaces Eqns 7 and 8, computing *g*_s_ from environmental data and plant hydraulic inputs with Eqns 4 and 5. To compute *A* from the *g*_s_ predicted by Eqn 4, we solved the limiting photosynthetic rates from the Collatz *et al*. (1991, 1992) model as functions of *c*_a_ and *g*_s_, as described in Notes S3.

### Leaf‐level SOX evaluation

We used a global compilation of leaf gas exchange data to evaluate the SOX capacity to reproduce leaf stomatal responses of a wide range of woody plants. This dataset contains observations compiled by Lin *et al*. (2015), complemented with other published and unpublished data (see Table S1 and Fig. S2 for additional information). In total, there are 3597 measurements of *g*_s_ and Ψ_pd_ together with environmental variables used for driving the model, that is, incident photosynthetic active radiation (*I*_par_), air temperature (*T*_a_), *c*_a_ and *D*. These data come from 30 woody plant species collected in 15 sites around the world (Fig. S2b). The Ψ_pd_ was measured on the same day as *g*_s_, and the environmental data was measured simultaneously with *g*_s_. The dataset included field and glasshouse observations, with environmental conditions varying from well‐watered to extreme drought (Ψ_pd_ = −7 MPa). These observations were grouped into the global plant functional type (PFT) categories from Harper *et al*. (2016) (Table 1). Harper *et al*. (2016) divides angiosperm tree species into broadleaf evergreen tropical trees (BET‐Tr), broadleaf evergreen temperate trees (BET‐Te) and broadleaf deciduous trees (BDT), while gymnosperms tree species are divided into needleleaf evergreen trees (NET) and needleleaf deciduous trees (NDT). Shrub species were divided into evergreen shrubs (ESh) and deciduous shrubs (DSh), and two grass PFTs defined by their photosynthetic pathway (C_3_ and C_4_). The grass PFTs and the NDT were excluded from the leaf‐level evaluation because no stomatal conductance data were available for these PFTs in the dataset used in this study.

**Table 1:** Residual sum of squares (RSS), number of leaf‐level stomatal conductance observations (*N*) used to fit *n* parameters to the data, and the resulting Akaike information criterion differences (ΔAIC) between stomatal optimization based on xylem hydraulics (SOX) and the *β*‐function

PFT	*N*	SOX		*β*‐function		ΔAIC
		RSS	*n*	RSS	*n*	
BET‐Tr	434	4.83	3	6.53	2	−126.1
BET‐Te	1334	19.68	3	37.37	2	−853.2
BDT	71	3.48	3	3.04	2	11.6
NET	1571	0.65	3	2.29	2	−1926.4
ESh	133	3.37	3	7.94	2	−112
DSh	64	2.76	3	8.03	2	−66.4

PFT, plant functional type; BET‐Tr, broadleaf evergreen tropical tree; BET‐Te, broadleaf evergreen temperate tree; BDT, broadleaf deciduous tree; NET, needleleaf evergreen tree; ESh, evergreen shrubs; DSh, deciduous shrubs.

The plant hydraulic parameters used in SOX (i.e. Ψ_50_, *a*, and *r*_pmin_) were fitted to the *g*_s_ data using an algorithm that minimizes the model residual sum of squares within the constraints of the observed Ψ_50_, *a* and *r*_pmin_. We compiled hydraulic data for each PFT from the literature to constrain the leaf‐level model fit. The Ψ_50_ for woody plants was obtained from a version of the Choat *et al*. (2012) dataset updated recently by Mencuccini *et al*. (2019b). The shape parameter *a* of the xylem vulnerability function (Eqn 2) was estimated from the linear gradient between Ψ_50_ and the Ψ when the plant loses 88% of its maximum hydraulic conductance. The *r*_pmin_ was estimated from branch‐level hydraulic conductivity measurements scaled from branch to whole plant, taking into account plant height, Huber value and xylem tapering using the calculations described in Christoffersen *et al*. (2016) and Savage *et al*. (2010) (Notes S4). All the data used for these calculations were obtained from the hydraulic dataset from Mencuccini *et al*. (2019b). We note that scaling branch to whole tree *r*_pmin_ requires several assumptions about tree hydraulic architecture (Notes S4). Therefore, the presented values of *r*_pmin_ must be considered as a reference useful only to assess if the *r*_pmin_ input values used in the model are within the same order of magnitude of the observations. The other parameters of the photosynthesis model used in SOX (Notes S3) were set equal to those in Harper *et al*. (2016).

The model predictive skill was evaluated using the model root‐mean‐square errors (RMSE) and the Nash & Sutcliffe (1970) model efficiency index (NSE). The NSE varies from −∞ to 1, with 1 indicating perfect agreement between model and observations, while NSE < 0 indicates that the mean value of the observations is a better predictor than the model. The model parsimony was evaluated using the Akaike information criterion (AIC), which penalizes model overparameterization (Bozdogan, 1987). We compared SOX AIC score with the *β*‐function (Eqns 7, 8). The parameters *f*_0_ and *D*_crit_, (Eqn 8) were fitted to the PFT *g*_s_ data, while *θ*_c_ and *θ*_w_ were held at their default values (−0.033 and − 1.5 MPa, respectively).

The uncertainty in plant hydraulic parameters caused by within‐PFT hydraulic variability was propagated to the model predictions using bootstrapped 95% confidence intervals. We created the interval based on 1000 model runs with parameters resampled from the hydraulic trait data for each PFT.

### Eddy‐covariance based JULES‐SOX evaluation

We evaluated default JULES and JULES‐SOX against daily GPP estimates derived from eddy flux tower data at 62 FLUXNET sites (http://fluxnet.fluxdata.org, Baldocchi *et al*., 2001) and eight LBA sites (https://daac.ornl.gov/LBA, Saleska *et al*., 2013), covering all the major biomes of the world (Fig. S2; Table S2). In 10 of these sites we also had data for surface (5–15 cm) soil moisture content, which was used to evaluate the model soil moisture dynamics predictions. We classified the land cover on each site using the International Geosphere‐Biosphere Programme (IGBP) classification (Loveland *et al*., 2000). Each site was classified as one of the following categories according to its prescribed PFT cover (Table S2): cropland (CRO), deciduous broadleaf forests (DBF), deciduous needleleaf forests (DNF), temperate evergreen broadleaf forests (EBF‐Te), tropical evergreen broadleaf forests (EBF‐Tr), evergreen needleleaf forest (ENF), grassland (GRA), mixed forest (MF), savannah (SAV), shrubland (SHR) and wetlands (WET). We grouped the IGBP categories open and closed shrublands into SHR, as we only had a single closed shrubland site. Similarly, woody savannah was grouped with SAV, as we only had two woody savannah sites. We divided the evergreen broad leaf forests category into EBF‐Te and EBF‐Tr, as these sites were dominated by distinct PFTs (BET‐Te and BET‐Tr, respectively).

We evaluated JULES‐SOX using the SOX hydraulic parameters (i.e. Ψ_50_, *a*, and *r*_pmin_) that minimized the residual sum of squares between SOX predictions and the eddy flux GPP observations from a subset of the sites used for model evaluation (Fig. S2; Table S2). Each site was used to calibrate the hydraulic parameters for its dominant PFT (i.e. the PFT covering > 50% of the site area), except for DSh, which was not dominant in any of the available sites. We used a site with DSh cover of 35% (US‐SRM) to calibrate the hydraulic parameters of this PFT. The hydraulic parameters of the others PFTs (if any) present on the site were kept constant during the model runs for parameter calibration. Similar to the leaf‐level evaluation, the parameter calibration in JULES‐SOX was constrained within the range of the observed values of Ψ_50_, *a*, and *r*_pmin_ for all PFTs, except for NDT, which did not have enough observations to satisfactorily constrain the model parameters. The Ψ_50_ for grasses was obtained from the Lens *et al*. (2016) dataset updated with data from Ocheltree *et al*. (2016).

### Model setup

The JULES and JULES‐SOX configurations used in this study employed the 10‐layer canopy scheme with sunlit and shaded leaves in each layer as described in Clark *et al*. (2011). The canopy radiation profile was given by the two‐stream approach from Sellers (1985), with the sun‐fleck penetration scheme from Mercado *et al*. (2009), and an exponential decrease of photosynthetic capacity through the canopy (Mercado *et al*., 2007). All the model runs used in this study were site‐level simulations driven with hourly local meteorological data. Vegetation dynamics (Cox, 2001) was turned off and the site PFT coverage by site was prescribed based on the site vegetation description obtained from the site principal investigators (Table S2) or information from the site available on the FLUXNET website (https://fluxnet.fluxdata.org/sites/site-list-and-pages/). Site soil hydraulic properties were parameterized using Brooks & Corey (1964) relations. These properties were derived from data collected at each site or, when local data were not available, calculated from the sand/silt/clay fractions in the nearest gridbox in the high‐resolution input file to the Met Office Central Ancillary Program (Dharssi *et al*., 2009), using approximations from Cosby *et al*. (1984). The model was spun up by recycling the meteorological data at each site for up to 50 yr.

## Results

### SOX sensitivity to environmental and hydraulic drivers

**Figure 1:**
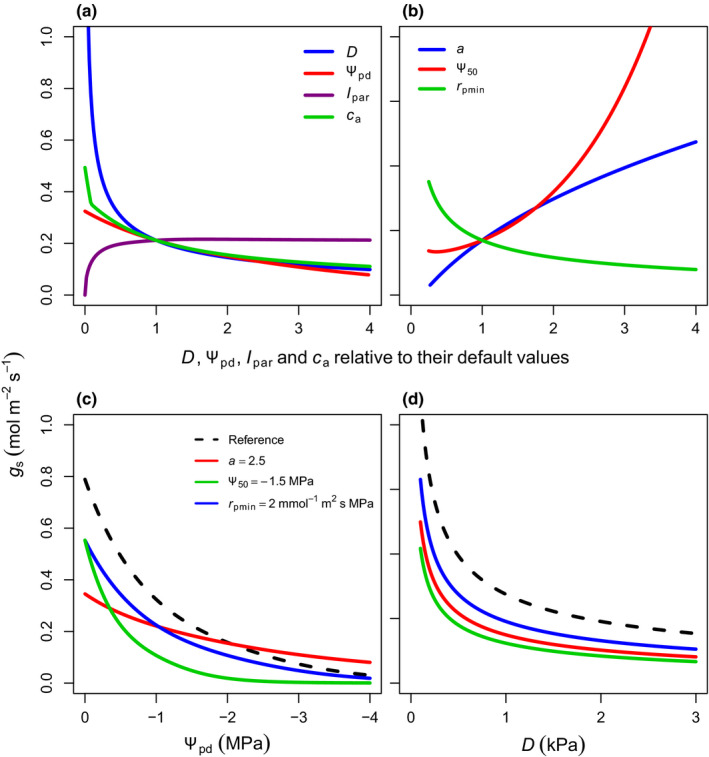
(a, b) Stomatal conductance (*g*_s_) sensitivity to environmental drivers (a) and plant hydraulic traits (b) as modelled by stomatal optimization based on xylem hydraulics (SOX) (*D*, vapour pressure deficit; Ψ_pd_, predawn water potential; *I*_par_, incident photosynthetically active radiation; *c*_a_, atmospheric CO_2_ partial pressure; Ψ_50_, Ψ when plant loses 50% of its maximum conductance; *a*, shape of vulnerability function; *r*_pmin_, minimum plant hydraulic resistance). Variables were changed individually while the others were held constant at their reference values (*D* = 0.5 kPa, Ψ_pd_ = −0.5 MPa, *I*_par_ = 600* *µmol m^−2^ s^−1^, *c*_a_ = 36 Pa, Ψ_50_ = −2 MPa, *a* = 3, *r*_pmin_ = 1 m^2^ s MPa mmol^−1^). For (c) and (d) the reference lines (dashed black) represent values of Ψ_50_ = −3 MPa, *a* = 5, *r*_pmin_ = 1 mmol^−1^ m^2^ s MPa, and the coloured lines show how changing each hydraulic parameter affects *g*_s_ response to Ψ_pd_ and *D*. In (c) and (d), *I*_par_ was set to 2000* *µmol m^−2^ s^−1^. The Rubisco maximum carboxylation rate at 25°C (*V*_cmax25_) was set to 100 µmol m^−2^ s and the rest of the photosynthetic parameters follow the broadleaf evergreen tropical tree (BET‐Tr) parameterization from Harper *et al*. (2016).

The SOX analytical approximation (Eqns 4, 5) has *g*_s_ responses to climate which are consistent with the patterns commonly reported in the literature (Mott, 1988; Leuning, 1995; Dewar *et al*., 2018). The *g*_s_ responses to *I*_par_ and *c*_a_ in SOX (Fig. 1a) are given by the ∂*A*/∂*c*_i_ gradient decreasing at low light because of the changes in the light response curve, as *A* starts being limited by light (Notes S3), or at high *c*_a_ (Notes S2). SOX correctly predicted stomatal closure in response to increased *c*_a_ under Rubisco‐limited conditions (Mott, 1988; Fig. 1a). The classical exponential *g*_s_ responses to *D* (Leuning, 1995) was reproduced in SOX (Fig. 1a) through the *D* effect on *ξ* (Eqn 5; Fig. S1a). An exponential *g*_s_ decline was also predicted by SOX in response to decreasing Ψ_pd_ (Fig. 1a), which summarizes both the responses to the soil water availability in the root zone and the hydraulic stress of transporting water to the top of the canopy (Eqn S1.2 in Notes S1). The plant hydraulic parameters modulated the model sensitivity to *D* or Ψ_pd_ (Fig. 1b–d), with a less negative Ψ_50_ or a higher *r*_pmin_ increasing the *g*_s_ sensitivity to Ψ_pd_ and *D* (Fig. 1c,d). The effect of the vulnerability curve shape parameter *a* was more complex: lower *a* increased *g*_s_ sensitivity to less negative Ψ_pd,_ but decreased *g*_s_ sensitivity to very negative Ψ_pd_ values (Fig. 1c).

The patterns produced by the analytical SOX were similar to the numerical version from Eller *et al*. (2018b), with a correlation coefficient ranging from 0.92 to 1 (Fig. S3). However, the use of linear gradients in Eqns 4 and 5 (Notes S2) can cause discrepancies between the different model versions under certain ranges of environmental conditions. The analytical version of SOX underestimated *g*_s_ at low *D* (Fig. S3), overestimated *g*_s_ at low *c*_a_, and *g*_s_ increased faster in response to light (Fig. S3) than in the numerical model.

### SOX leaf‐level evaluation

**Figure 2:**
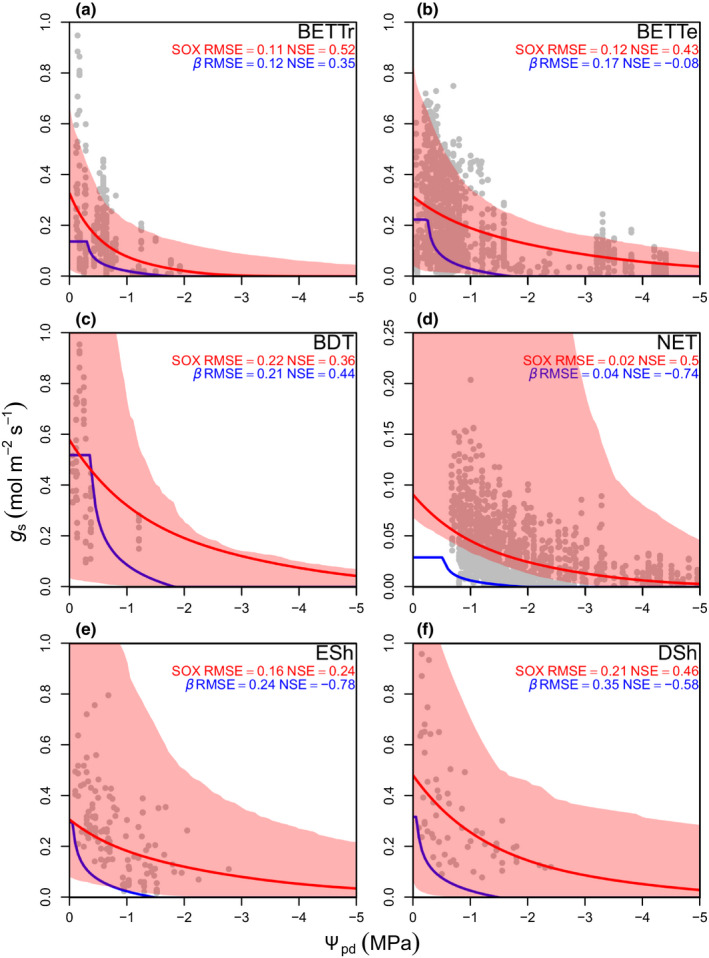
Predicted and observed (grey points) stomatal conductance (*g*_s_) response to changes in leaf predawn water potential (Ψ_pd_) for the woody plant functional types (PFT) from Harper *et al*. (2016), except for needleleaf deciduous trees, which were not present in the dataset used in this study. The red and blue lines are the best fits from the stomatal optimization based on xylem hydraulics (SOX) and *β*‐function (Eqns 7, 8), respectively. The shaded regions are nonparametric 95% confidence boundaries derived from 1000 bootstrapping replications of the SOX hydraulic inputs. All environmental conditions except Ψ_pd_ were held constant at their median values when the *g*_s_ measurements were taken. The Ψ_pd_ was converted in soil volumetric water content to drive the *β*‐function using the Brooks & Corey (1964) equations parameterized with soil physical properties derived from the Met Office Central Ancillary Program (Dharssi *et al*., 2009). The model fit to data is shown as the root‐mean‐square errors (RMSE) and Nash‐Sutcliffe (1970) model efficiency index (NSE). The PFT abbreviations in each panel are as follows: (a) broadleaf evergreen tropical tree (BET‐Tr); (b) broadleaf evergreen temperate tree (BET‐Te); (c) broadleaf deciduous tree (BDT); (d) needleleaf evergreen tree (NET); (e) evergreen shrubs (ESh); and (f) deciduous shrubs (DSh).

Stomatal optimization based on xylem hydraulics simulated the observed leaf‐level *g*_s_ responses to soil drought better than the *β*‐function in all the studied woody PFTs, except BDT (Fig. 2). The *β‐*function predicted that all PFTs will reach *g*_s_ = 0 at Ψ_pd_ > −2 MPa, whereas SOX predicted *g*_s_ > 0 even when Ψ_pd_ < −4 MPa in some PFTs (Fig. 2b,e). The less conservative stomatal behaviour predicted by SOX produced a NSE that was, on average, 0.65 higher and a RMSE that was 26% lower than the *β‐*function. Most of the observed *g*_s_ was within SOX 95% confidence bounds derived from the hydraulic parameters’ uncertainty (shaded region in Fig. 2). The only values outside SOX uncertainty boundaries were the highest *g*_s_ values in BET‐Tr and BET‐Te (Fig. 2a,b), and the lowest NET *g*_s_ values when Ψ_pd_ > −3.5 MPa (Fig. 2d).

Stomatal optimization based on xylem hydraulics produced a better fit to the *g*_s_ data, which resulted in a lower AIC than the *β‐*function for all PFTs, except BDT (Table 1). Fitting the two empirical parameters of the Jacobs (1994) equation (*f*_0_ and *D*_crit_; Eqn 8) to the *g*_s_ data results in a *β*‐function AIC score that is 512.1 higher than SOX (Table 1). For the BDT observations, the *β*‐function results in an AIC score that is 11.6 lower than SOX. Our BDT observations were restricted to relatively well‐watered conditions (lowest Ψ_pd_ was − 1.2 MPa), which limits the utility of this dataset to evaluate the model responses to soil drought.

### JULES‐SOX site‐level calibration

The hydraulic parameters that maximized the JULES‐SOX fit to the GPP data at the calibration sites (Table S2; Fig. S2) were within 1 SD of the mean observed hydraulic parameters for most PFTs (Table 2). The gymnosperm PFTs (NDT and NET) required Ψ_50_ values 1.6 MPa less negative than their observed Ψ_50_ means to fit the GPP data, which is lower than the observed SD range but still within the range of Ψ_50_ observations for NET (Ψ_50_ ranges from − 2.3 to − 7.5 MPa in NET). The NDT and BET‐Tr calibrated *a* were also slightly lower than the SD range (Table 2), but within the observed *a* range for BET‐Tr (*a* ranges from 1.8 to 7.8 in BET‐Tr). The only PFT with a calibrated *r*_pmin_ outside the SD range of the mean *r*_pmin_ was ESh (Table 2).

**Table 2:** Observed (Obs) mean (± SD) hydraulic parameters compiled from the literature for each plant functional type (PFT) from JULES (Harper *et al*., 2016)

PFT	Ψ_50_ (MPa)			*a* (unitless)			*r*_pmin_ (10^−3^ mol^−1^ m^2^ s MPa)		
	*N*	Obs	Cal^a^	*N* b	Obs	Cal	*N*	Obs	Cal
BET‐Tr	77	−1.9 (± 1.3)	−1.7	20	4.4 (± 2.1)	2.1	40	2.2 (± 3.4)	0.6
BET‐Te	44	−2.7 (± 1.5)	−1.8	17	3.7 (± 1.8)	2.8	40	3.1 (± 8)	5
BDT	87	−2.6 (± 1.4)	−1.6	43	5.5 (± 3.8)	3.5	31	5.3 (± 5.6)	0.5
NET	48	−4.2 (± 1.2)	−2.6	25	8.7 (± 4.9)	4.9	20	2.4 (± 1.8)	4.2
NDT	5	−3.4 (± 0.6)	−1.8	2	7.4 (± 5)	1.8	2	8 (± 4.3)	9
C_3_	45	−3.1 (± 1.6)	−2.4	–	–	2.2	–	–	3.2
C_4_	15	−2.7 (± 1.7)	−1.5	–	–	1.8	–	–	9.5
ESh	61	−4 (± 2.2)	−2.1	53	4.1 (± 3.3)	2.5	49	1.5 (± 1.8)	9.5
DSh	26	−4 (± 2.3)	−1.8	3	3.4 (± 2.2)	2.1	4	2.6 (± 2.4)	5

BET‐Tr, broadleaf evergreen tropical tree; BET‐Te, broadleaf evergreen temperate tree; BDT, broadleaf deciduous tree; NET, needleleaf evergreen tree; NDT, needleleaf deciduous tree; C_3_, C_3_ grasses; C_4_, C_4_ grasses; ESh, evergreen shrubs; DSh, deciduous shrubs.

a The calibrated (Cal) columns are the parameter values that maximize the fit of the Joint UK Land Environment Simulator–stomatal optimization based on xylem hydraulics (JULES‐SOX) to observed gross primary productivity (GPP) in the calibration sites (see Supporting Information Table S2; Fig. S2).

b The *N* column is the number of species compiled for the correspondent parameter.

**Figure 3:**
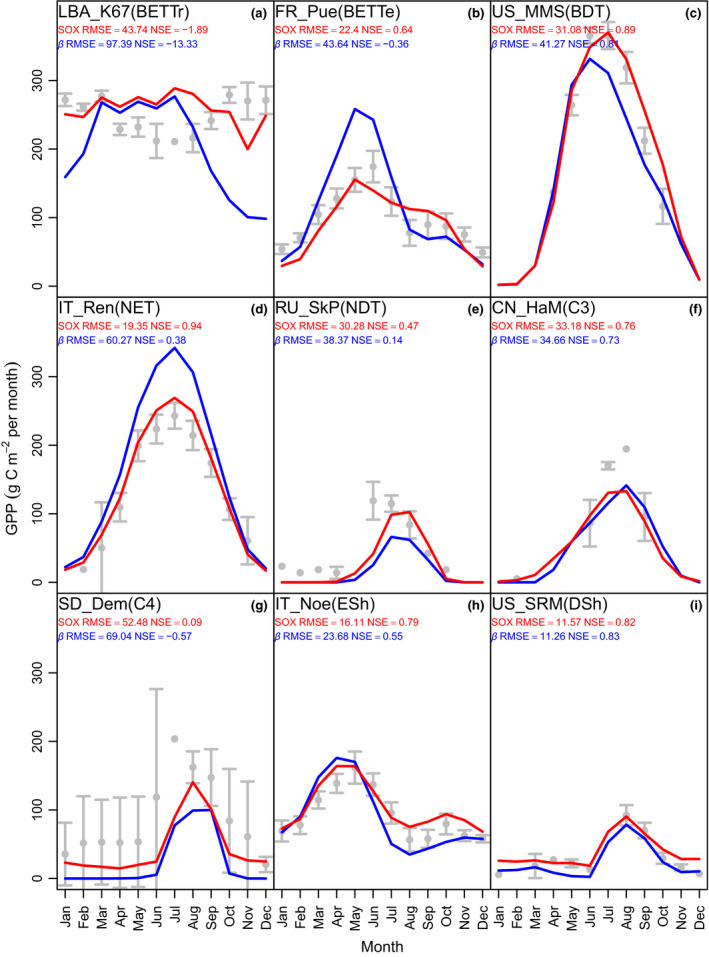
Monthly mean gross primary production (GPP) modelled by default Joint UK Land Environment Simulator (JULES, blue line) and JULES‐stomatal optimization based on xylem hydraulics (JULES‐SOX, red line) vs observations (grey points are means and bars are 2 × SE) at each eddy flux site used for calibrating the SOX hydraulic parameters (plant functional type (PFT); Supporting Information Table S2; Fig. S3). The model fit to data is shown as the root‐mean‐square errors (RMSE) and Nash‐Sutcliffe (1970) model efficiency index (NSE). The PFT abbreviations in each panel are as follows: (a) broadleaf evergreen tropical tree (BET‐Tr); (b) broadleaf evergreen temperate tree (BET‐Te); (c) broadleaf deciduous tree (BDT); (d) needleleaf evergreen tree (NET); (e) needleleaf deciduous tree (NDT); (f) C_3_ grasses (C_3_); (g) C_4_ grasses (C_4_); (h) evergreen shrubs (ESh); (i) deciduous shrubs (DSh).

The monthly GPP modelled by JULES‐SOX fitted the eddy covariance GPP data better than the default JULES in eight out of the nine sites used for parameter calibration (Table S2; Figs 3, S2). The default JULES NSE was 0.01 higher in the DSh site (Fig. 3i), whereas in all the other sites JULES‐SOX had a better fit. The difference between JULES‐SOX and default JULES NSE ranged from 0.03 for C3 grasses (Fig. 3f) to 11.44 for BET‐Tr (Fig. 3a). The large improvement in the BET‐Tr site was caused by the lower GPP decline predicted by SOX during January–March and September–December. The decline in BET‐Tr GPP in default JULES can be attributed to the *β*‐factor overestimating the effects of soil moisture on the vegetation carbon assimilation during drier periods (Fig. S4a). On average, JULES‐SOX NSE for GPP was 1.59 higher than default JULES, while its RMSE was 38% lower than JULES.

**Figure 4:**
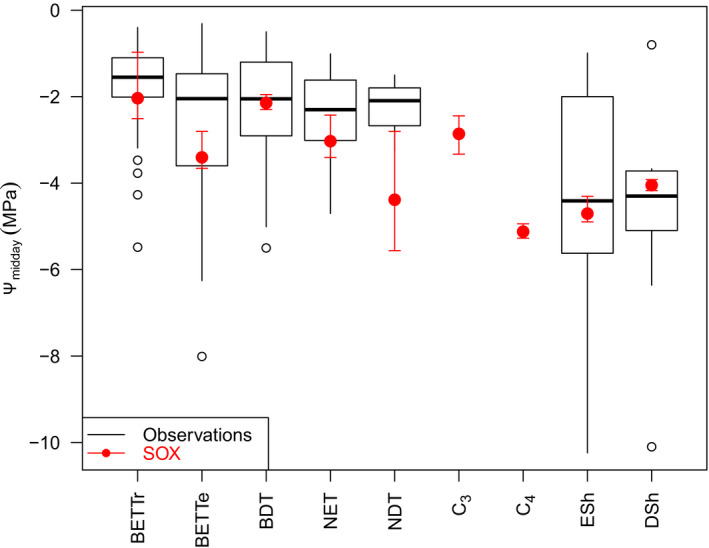
Minimum observed midday leaf water potential (Ψ_midday_) from 279 woody plant species compiled from the literature grouped using the Harper *et al*. (2016) plant functional type (PFT) categories. The Ψ_midday_ for each of the calibration sites as modelled by stomatal optimization based on xylem hydraulics (SOX) (see Supporting Information Table S2; Fig. S2) is plotted in red. The circle is the mean Ψ_midday_ and the arrows indicate the minimum and maximum Ψ_midday_. The data for the deciduous PFT were restricted to the growing season. The PFT abbreviations in each panel are as follows: broadleaf evergreen tropical tree (BET‐Tr); broadleaf evergreen temperate tree (BET‐Te); broadleaf deciduous tree (BDT); needleleaf evergreen tree (NET); needleleaf deciduous tree (NDT); C_3_ grasses (C_3_); (g) C_4_ grasses (C_4_); evergreen shrubs (ESh); deciduous shrubs (DSh).

The less conservative stomatal behaviour predicted by SOX resulted in higher evapotranspiration rates throughout the year (Figs S5, S6), which depleted soil moisture to lower values than the *β‐*function in default JULES during drier periods (Figs S4, S7). The soil moisture dynamics from JULES‐SOX are more closely aligned with the monthly soil moisture observations in eight out of the 10 sites where soil moisture data were available (Fig. S7). JULES‐SOX NSE for monthly soil moisture was 1.67 higher and RMSE was 19% lower than default JULES. JULES‐SOX also simulates realistic Ψ_c_ for most PFTs (Figs 4, S4). The modelled Ψ_c_ at the calibration sites is within the interquartile range of the observed minimum Ψ_c_ at midday for all woody PFTs, except NDT (Fig. 4).

### Biome‐level JULES‐SOX evaluation

**Figure 5:**
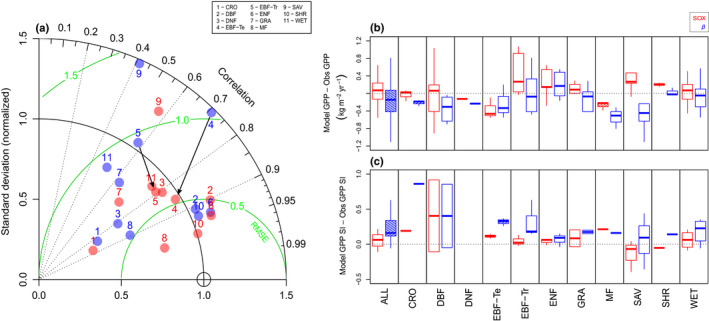
(a) The Taylor diagram shows the difference in Joint UK Land Environment Simulator (JULES) and JULES‐stomatal optimization based on xylem hydraulics (JULES‐SOX) skill to predict the monthly gross primary productivity (GPP) in each studied biome. Green lines are the model‐centred root‐mean‐square errors (RMSE), and points closer to the reference circle on the *x*‐axis indicate higher model skill. The two arrows highlight the improvement in model skill for tropical evergreen broadleaf forests (EBF‐Tr) and temperate evergreen broadleaf forests (EBF‐Te). The boxplot panels show the differences between models (default JULES in blue, JULES‐SOX in red) and observations (Obs) in the annual GPP (b) and the GPP seasonality (GPP SI) (c). Data gaps were excluded from the annual GPP calculations for both models and observations, and therefore the differences can be used to evaluate the model skill, but the absolute values do not represent the total annual GPP in each biome. The GPP SI was computed using the approach from Walsh & Lawler (1981). Boxes filled with lines are different (at *α* = 0.05) from 0 in a one‐sample *t*‐test. The biomes are abbreviated as follows: cropland (CRO); deciduous broadleaf forests (DBF); deciduous needleleaf forests (DNF); temperate evergreen broadleaf forests (EBF‐Te); tropical evergreen broadleaf forests (EBF‐Tr); evergreen needleleaf forest (ENF); grassland (GRA); mixed forest (MF); savannah (SAV); shrubland (SHR); and wetlands (WET).

Using JULES‐SOX with calibrated SOX hydraulic parameters produced a better fit to the GPP data than default JULES for 50 out of the 70 eddy flux evaluation sites (Tables 3, S2; Fig. 5). Across all biomes the JULES‐SOX median NSE was 0.19 higher than default JULES, and its RMSE was 19% lower (Table 3). The difference between JULES‐SOX and JULES skill was highest at EBF‐Tr sites, which have a median NSE 3.18 higher and RMSE 45% lower in JULES‐SOX (Table 3; Fig 5a). The fit of EBT‐Te to data was also improved substantially by JULES‐SOX, with JULES‐SOX having a median NSE 1.01 higher and RMSE 18% lower (Fig. 5a; Table 3). Default JULES only outperformed JULES‐SOX at CRO, which had a median NSE 0.08 lower in JULES‐SOX, and GRA, where the RMSE 5% was higher in JULES‐SOX (Fig. 5a; Table 3).

**Table 3:** Median Nash‐Sutcliffe (1970) model efficiency index (NSE) and root‐mean‐square error (RMSE) for the biomes used for evaluating the Joint UK Land Environment Simulator–stomatal optimization based on xylem hydraulics (JULES‐SOX) and the default JULES

Biome^a^	*N* b	JULES‐SOX		JULES	
		NSE	RMSE	NSE	RMSE
CRO	3	0.49	123.12	0.57	141.1
DBF	7	0.89	37.32	0.83	47.19
DNF	1	0.58	25.93	0.37	31.97
EBF‐Te	3	−0.23	45.22	−1.24	66.36
EBF‐Tr	6	0.41	40.36	−2.77	73.53
ENF	5	0.9	34.14	0.59	40.58
GRA	12	0.22	32.31	−0.01	30.62
MF	3	0.85	47.87	0.59	79.29
SAV	5	−0.4	59.72	−2.12	89.69
SHR	4	0.78	14.90	0.64	15.92
WET	21	0.68	32.23	0.46	38.67

a Biome abbreviations are as follows: CRO, cropland; DBF, deciduous broadleaf forests; DNF, deciduous needleleaf forests; EBF‐Te, temperate evergreen broadleaf forests; EBF‐Tr, tropical evergreen broadleaf forests; ENF, evergreen needleleaf forest; GRA, grassland; MF, mixed forest; SAV, savannah; SHR, shrubland; WET, wetlands.

b The *N* column is the number of sites representing the biome in the eddy covariance dataset.

Default JULES significantly underestimated the observed mean annual GPP by 143.3 g C m^−2^ across all biomes, which corresponds to 13.6% of the observed mean annual GPP (Fig. 5b). JULES‐SOX deviation from the observed mean annual GPP was considerably smaller (71.6 g C m^−2^; Fig. 5b). The significantly lower annual GPP predicted by default JULES can be attributed to *β‐*function‐induced GPP declines, which also produced a stronger GPP seasonality than is present in the data (Fig. 5c). JULES overestimated the median observed GPP seasonality by 70%, compared with a 13% overestimation by JULES‐SOX (Fig. 5c). This difference means JULES predicts 17% of the sites have a markedly seasonal GPP with a Seasonality Index (SI; Walsh & Lawler, 1981) higher than 0.8, while just 4% of the sites actually have SI > 0.8. JULES‐SOX predicts only 8% of the sites would have SI > 0.8.

**Figure 6:**
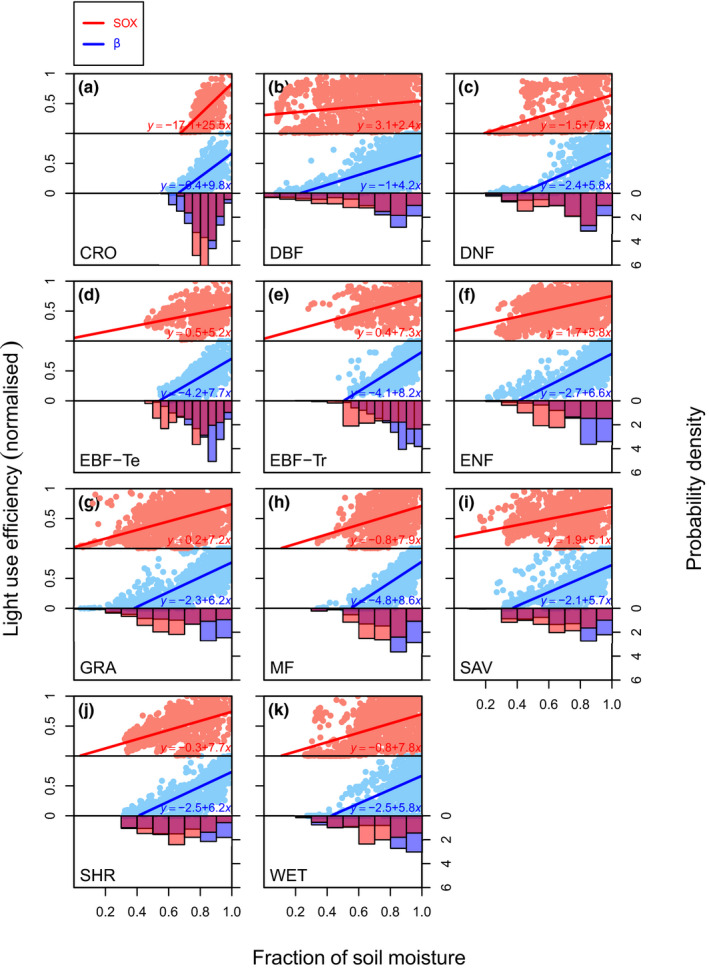
Model predictions of the normalized light‐use efficiency responses to soil moisture, expressed as a fraction of the soil moisture saturation point at the top 1 m of soil. The light use efficiency is the ratio between gross primary productivity and the photosynthetic active radiation absorbed by the canopy. The default JULES predictions are in blue and JULES‐SOX predictions in red. The lines in the scatter plot panels are linear regressions fit to the data. The histograms on the bottom panels are the soil moisture probability density predicted by each model. The biomes are abbreviated as follows: (a) cropland (CRO); (b) deciduous broadleaf forests (DBF); (c) deciduous needleleaf forests (DNF); (d) temperate evergreen broadleaf forests (EBF‐Te); (e) tropical evergreen broadleaf forests (EBF‐Tr); (f) evergreen needleleaf forest (ENF); (g) grassland (GRA); (h) mixed forest (MF); (i) savannah (SAV); (j) shrubland (SHR); and (k) wetlands (WET).

The light‐use efficiency (LUE; Fig. 6) is the ratio between GPP and the *I*_par_ absorbed by the canopy (Stocker *et al*., 2018), and can be used to disentangle the effects of soil moisture and light availability controlling the vegetation GPP. The JULES LUE declined as soil dried out, with a mean linear slope of 1.21 (± 0.1) across all biomes. By contrast, the JULES‐SOX LUE–soil moisture relationship had a mean slope of 0.73 (± 0.21), with some biomes, such as DBF, reaching a slope as low as 0.22 (Fig. 6b). The consequence of sustaining higher LUE at low soil moisture in JULES‐SOX is a greater depletion of soil moisture, as indicated by the more left‐skewed soil moisture probability distribution predicted by JULES‐SOX (lower panels in Fig. 6). The mean moisture content of the top 1 m of soil predicted by JULES‐SOX was, on average, 10% lower than default JULES. In JULES‐SOX some biomes, such as ENF, could reach a soil moisture, on average, 17% lower than JULES (Fig. 6f).

## Discussion

We report the first evaluation of a LSM using a stomatal optimization model fully based on xylem hydraulics to drive the vegetation stomatal responses to climate. Our results provide support for the SOX underlying hypothesis that stomata evolved to balance carbon assimilation with instantaneous hydraulic conductance loss. The risk of mortality through hydraulic failure (Choat *et al*., 2012; Rowland *et al*., 2015; Anderegg *et al*., 2016; Adams *et al*., 2017) should drive the evolution of mechanisms to prevent the plant from reaching lethal embolism thresholds (Sperry, 2004). There is abundant evidence that stomata controls xylem tension, and consequently embolism (Hubbard *et al*., 2001; Brodribb *et al*., 2003; Meinzer *et al*., 2009; Klein, 2014). Our model represents this xylem–stomata coordination through the assumption of optimization by natural selection (Wolf *et al*., 2016).

Whereas our model fits the observations of most PFTs better than its empirical alternative, there is still a considerable amount of unexplained variance in the data (Fig. 2). This can be partially attributed to the large hydraulic heterogeneity within each PFT, but we must also acknowledge that many processes not directly related to xylem hydraulics are important to plant life history and stomatal evolution. Processes related to nutrient use and acquisition, carbohydrate allocation and storage, the maintenance of tissues and biochemical apparatus, and protection from pathogens and herbivores (Melotto *et al*., 2008; Cramer *et al*., 2009; Prentice *et al*., 2014) could all explain part of our model residual variance. It is extremely important to explore the relevance of these processes in future research on stomatal optimality. However, the SOX model as we propose it already provides a parsimonious alternative to the empirical models commonly used in LSMs.

Our findings that xylem hydraulics‐based models can adequately simulate stomatal behaviour agree with other recent studies. For example, Anderegg *et al*. (2018b) shows that a hydraulics‐based optimization model can simulate the stomatal behaviour of woody plants better than the CF model. More recently, Wang *et al*. (2019) shows that a similar hydraulics‐based model can predict stomatal responses to increased CO_2_ better than the Ball–Berry–Leuning empirical model (Leuning, 1995). These results show the potential of using plant hydraulics to model the stomatal behaviour of plants across contrasting environmental conditions, and supports its use in ESMs to project the evolution of global climate.

The analytical formulation developed for SOX facilitates its coupling to LSMs, allowing the host LSM to constrain its predictions using plant hydraulic information. We show that inclusion of plant hydraulics in JULES through SOX improves its capabilities to simulate GPP and soil moisture dynamics in most of the studied biomes (Figs 3, 4, 5). In addition, SOX opens new possibilities to evaluate LSM predictions and expands the range of hypotheses that can be tested with JULES. Using JULES‐SOX within ESMs will allow us to understand how hydraulic processes affect climatic and biogeochemical cycles at the global scale, as well as to investigate the role of plant hydraulics on vegetation distribution and its response to climate change.

### SOX parametrization and parsimony

Other LSMs and DGVMs have already successfully employed principles of plant hydraulics (Hickler *et al*., 2006; Bonan *et al*., 2014; Kennedy *et al*., 2019), but JULES‐SOX is the first LSM to use the new generation of hydraulically based stomatal optimization models (Wolf *et al*., 2016; Sperry *et al*., 2017; Anderegg *et al*., 2018b; Eller *et al*., 2018b) to predict stomatal responses to climate. The SPA (Williams *et al*., 1996) adaptation to the community land model (CLM) by Bonan *et al*. (2014) was one of the first approaches to link plant stomatal function to plant hydraulic processes in a LSM. Despite SPA being an extremely useful model, SOX has an advantage in circumstances where assuming a strict isohydric behaviour is not appropriate (Klein, 2014; Martinez‐Vilalta *et al*., 2014). In relation to SOX, SPA does not represent dynamic changes in the plant hydraulic conductance or an anisohydric mode of stomatal regulation (Williams *et al*., 1996; Fisher *et al*., 2006). However, SPA accounts for plant hydraulic capacitance, which can be important for plant functioning, especially during the early morning (Goldstein *et al*., 1998), and is currently not implemented in SOX.

Recently, Kennedy *et al*. (2019) implemented a plant hydraulic scheme (PHS) in a CLM. The PHS simulates dynamic changes in hydraulic conductance in different compartments along the soil–atmosphere continuum, providing a more detailed representation than SOX of hydraulic processes occurring along the soil–plant hydraulic pathway. However, PHS still requires empirical parameters to represent stomatal responses to soil drought and *D* (Kennedy *et al*., 2019), namely the *g*_0_ and *g*_1_ parameters from the Medlyn *et al*. (2011) model, and the critical and wilting points used in the empirical stress factor. The main advantage of SOX is providing an alternative to the *β*‐function and empirical stomatal parameters by linking plant hydraulic processes directly to stomatal functioning. As we treat the soil–plant–atmosphere pathway as a single hydraulic compartment, SOX only requires the hydraulic parameters *r*_pmin_, Ψ_50_ and *a* to predict stomatal responses to climate. This makes SOX even more parsimonious than default JULES, which requires four empirical parameters to simulate stomatal responses to climate (Eqns 7, 8) and does not simulate any aspect of vegetation hydraulic functioning (Clark *et al*., 2011).

We show that the SOX hydraulic parameters in most PFTs can be constrained with plant branch‐level hydraulic observations (Table 2), which is an advantage over models that employ empirical parameters difficult to constrain and interpret biologically. However, we observed discrepancies between the SOX‐calibrated parameters and the observed hydraulic traits in certain PFTs (Table 2). In some cases, such as NDT, the parameter discrepancy may have been a result of a very restricted observational sampling of hydraulic parameters in this group. The NDT only had Ψ_50_ data for five species and *a* and *r*_pmin_ for two species (Table 2). Considering that the observations used in this study were not collected in the same FLUXNET sites used to evaluate SOX, some of the observed discrepancies between calibrated and measured parameters might reflect hydraulic differences between populations treated as the same PFT in this study. For example, the deciduous angiosperms species present in the XFT dataset used in this study contain mostly hydraulic data from cold‐deciduous temperate species (Mencuccini *et al*., 2019b), which might not be adequate to describe the hydraulic system of tropical and subtropical drought‐deciduous. Our hydraulic scheme opens up possibilities of improving the representation of different global vegetation types in JULES with different hydraulic and phenological strategies. Capturing the large diversity of ecological strategies in plants is important to simulate species‐rich ecosystems such as tropical forests (Xu *et al*., 2016).

Anderegg *et al*. (2018a) computed the community‐weighted average values for Ψ_50_ in two of the FLUXNET sites used in this study (US‐MMS and IT‐Ren) and obtained values closer to the calibrated values for BDT and NET (−2.1 and −3.6 MPa, respectively) than the means from our compiled hydraulic dataset (Table 2). In Eller *et al*. (2018b) a numerical version of SOX outperformed the *β‐*function approach when parameterized with locally measured branch‐level hydraulic data from EBF‐Tr. These findings suggest that SOX can be constrained with *in situ* hydraulic measurements when these are available. However, we must also consider the possibility that there are intrinsic limitations in using branch‐level hydraulic data to parameterize the model. Roots and leaves can be more vulnerable to embolism than branches (Bartlett *et al*., 2016; Wolfe *et al*., 2016), which can make these tissues bottleneck plant hydraulic conductance during drought. The soil outside the roots can also limit plant hydraulic conductance and, ultimately, control its water use (Fisher *et al*., 2007). These bottlenecks could bias the SOX‐calibrated hydraulic parameters towards the limiting component and explain its departure from the branch‐level hydraulic data. In this case, SOX parameterization would benefit from the use of more integrative methodologies to estimate hydraulic parameters that represent the entire soil–plant hydraulic vulnerability (Eller *et al*., 2018a). Alternatively, the SOX structure (i.e. the *K* function in Eqn 2) would need to explicitly represent the variability between different hydraulic compartments along the soil–plant–atmosphere pathway, similar to SPA or other models (Eller *et al*., 2018b; Kennedy *et al*., 2019; Mencuccini *et al*., 2019b).

### Ecosystem‐level implications of SOX

Stomatal optimization based on xylem hydraulics improved JULES GPP simulation in over 70% of the 70 studied sites, and soil moisture dynamics in 80% of the 10 sites where soil moisture data were available. This improved fit was achieved using hydraulic parameters calibrated against the GPP data of a small subset of eddy flux sites (the sites in Fig. S2), which suggests that the calibrated parameters are generic enough to be used in global simulations. The lower sensitivity of SOX to soil moisture improved the simulations of annual GPP (Fig. 5) and predicted terrestrial biomes to assimilate on average 2.58 Mg C ha^−1^ yr^−1^ or 30% more than predicted by default JULES. This increased carbon assimilation could affect Earth's atmospheric CO_2_ evolution and climate change projections (Cox *et al*., 2000; Winkler *et al*., 2019).

The JULES‐SOX model particularly improved the fit of EBF‐Tr sites to the observations (Fig. 5; Table 3), using hydraulic parameters very similar to those observed in BET‐Tr (Table 2). Considering that SOX is also able to capture the response of EBF‐Tr even to extreme experimental drought (Eller *et al*., 2018b), JULES‐SOX may contribute to decrease the large uncertainty in how these important ecosystems will respond to climate change (Sitch *et al*., 2008). Tropical forest productivity estimated by SOX is less sensitive to seasonal soil drought (Figs 3, S4), which is consistent with the little seasonality often observed in tropical forest–atmosphere CO_2_ exchange (Grace *et al*., 1995; Carswell *et al*., 2002; Alden *et al*., 2016), as well as to forest responses to experimental drought (Meir *et al*., 2009; da Costa *et al*., 2010; Meir *et al*., 2018). da Costa *et al*. (2018) showed that even after 15 yr of partial rainfall exclusion, Amazon trees can maintain or even increase their transpiration rates (albeit following significant mortality). Whereas tropical forest resistance to drought has previously been attributed only to deep roots possessed by the vegetation (Nepstad *et al*., 1994), our results indicate that plants more resistant to embolism could maintain their carbon assimilation during drought even without a deeper root system.

The unavoidable consequence of maintaining stomatal gas exchange during soil drought is a greater depletion of soil moisture reserves (Figs 6, S4, S7). This behaviour is a direct consequence of the main assumption in SOX, which reflects a ‘use or lose it’ stomatal regulation strategy with respect to soil moisture (Sperry *et al*., 2017). SOX assumes plants with a more conservative water‐use strategy will be outcompeted by neighbouring plants with a less conservative stomatal behaviour (Wolf *et al*., 2016). The demographic consequences of the stomatal regulation strategy embedded in SOX should be explored in future studies using the dynamic vegetation component of JULES (Cox, 2001; Moore *et al*., 2018). The more competitive soil moisture dynamics predicted by SOX, together with a more accurate representation of vegetation drought‐induced mortality, which also can be developed from SOX, might be the key to predicting sudden and widespread vegetation die‐off during droughts that have been increasingly reported in ecosystems around the globe (Allen *et al*., 2010; Worrall *et al*., 2010; Meir *et al*., 2015).

## Author contributions

CBE, LR, MM, SS and PMC led the scientific development of SOX. PMC and CBE derived the analytical solution. CBE evaluated leaf‐level SOX using data provided by LR, PM, MM, TR, BEM, YW, TK, GST, RSO, ISM, BHPR. CBE and KW coded SOX into JULES. KW and AH created a JULES suite used by CBE to evaluate JULES‐SOX against eddy covariance data collected by KF, GW, LM, among other FLUXNET and LBA PIs. All authors contributed to writing the manuscript.

## Supporting information

Please note: Wiley Blackwell are not responsible for the content or functionality of any Supporting Information supplied by the authors. Any queries (other than missing material) should be directed to the *New Phytologist* Central Office.

**Fig. S1** Responses of Eqns 4 and 5 to environmental drivers.
**Fig. S2** Maps of the observation sites used on this study.
**Fig. S3** Agreement between numerical and analytical SOX.
**Fig. S4** Daily drought evolution modelled by JULES and JULES‐SOX.
**Fig. S5** Differences between evapotranspiration predicted by JULES and JULES‐SOX.
**Fig. S6** Seasonal variation in modelled and observed evapotranspiration.
**Fig. S7** Seasonal variation in modelled and observed soil moisture.
**Notes S1** Analytical SOX derivation.
**Notes S2** Computing A and V numerical derivatives.
**Notes S3** Leaf photosynthesis model solved for stomatal conductance.
**Notes S4** Whole‐tree hydraulic conductance and xylem tapering calculations.
**Table S1** Details for the data used in the SOX leaf‐level evaluation.
**Table S2** Sites used for the ecosystem‐level evaluation of JULES‐SOX.
