# Supplementary material for: Stomatal optimization based on xylem hydraulics (SOX) improves land surface model simulation of vegetation responses to climate
Source: New Phytol. 2020 Feb 17;226(6):1622–37. doi: 10.1111/nph.16419 (PMC7318565; doi:10.1111/nph.16419)
Supplement: Supplementary file 1 — Fig. S1 Responses of Eqns 4 and 5 to environmental drivers. Fig. S2 Maps of the observation sites used on this study. Fig. S3 Agreement between numerical and analytical SOX. Fig. S4 Daily drought evolution modelled by JULES and JULES‐SOX. Fig. S5 Differences between evapotranspiration predicted by JULES and JULES‐SOX. Fig. S6 Seasonal variation in modelled and observed evapotranspiration. Fig. S7 Seasonal variation in modelled and observed soil moisture. Notes S1 Analytical SOX derivation. Notes S2 Computing A and V numerical derivatives. Notes S3 Leaf photosynthesis model solved for stomatal conductance. Notes S4 Whole‐tree hydraulic conductance and xylem tapering calculations. Table S1 Details for the data used in the SOX leaf‐level evaluation. Table S2 Sites used for the ecosystem‐level evaluation of JULES‐SOX. [file NPH-226-1622-s001.pdf]

***New Phytologist* Supporting Information**

Article title: Stomatal optimisation based on xylem hydraulics (SOX) improves land surface model simulation of vegetation responses to climate
Authors: Eller CB, Rowland L, Mencuccini M, Rosas T, Williams K, Harper A, Medlyn BE, Wagner Y, Klein T, Teodoro GS, Oliveira RS, Matos IS, Rosado BHP, Fuchs K, Wohlfahrt G, Montagnani L, Meir P, Sitch S, Cox PM
Article acceptance date: 03 January 2020

The following Supporting Information is available for this article:

Fig. S1 Responses of equations 4 and 5 to environmental drivers.

Fig. S2 Maps of the observation sites used on this study.

Fig. S3 Agreement between numerical and analytical SOX.

Fig. S4 Daily drought evolution modelled by JULES and JULES-SOX.

Fig. S5 Differences between evapotranspiration predicted by JULES and JULES-SOX.

Fig. S6 Seasonal variation in modelled and observed evapotranspiration.

Fig. S7 Seasonal variation in modelled and observed soil moisture.

Table S1 Details for the data used on the SOX leaf-level evaluation

Table S2 Sites used for the ecosystem-level evaluation of JULES-SOX

Notes S1 Analytical SOX derivation

Notes S2 Computing A and V numerical derivatives

Notes S3 Leaf photosynthesis model solved for stomatal conductance

Notes S4 Whole tree hydraulic conductance and xylem tapering calculations

Fig. S1 Responses of equation 5 (a) and 4 (b) to changes in environmental conditions. The ξ decreases at lower pre-dawn water potential (Ψ_{pd}), due to the steeper gradient of the xylem vulnerability function ($\partial K/\partial \Psi_m$ $1/K$) and at higher vapour-pressure deficit (D). Lower ξ results in lower g_s (i.e. stomatal closure) in equation 4 (b), which also responds to changes in light or atmospheric CO2 through the $\partial A/\partial c_i$ gradient changes.

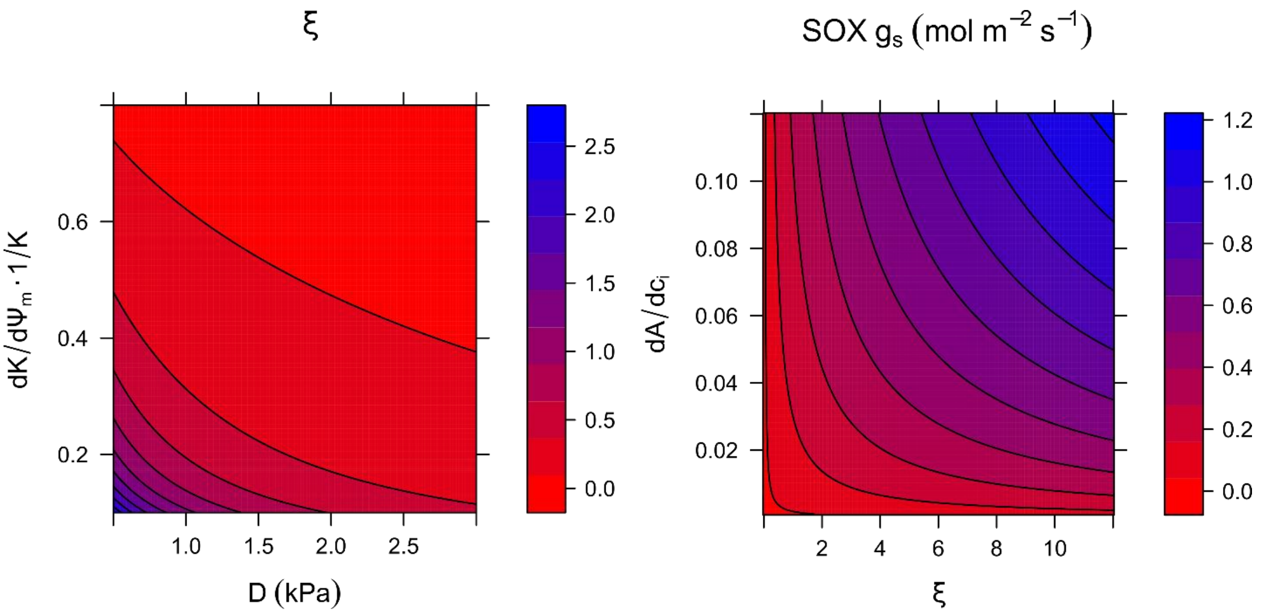

Fig. S2 Map of the eddy flux sites used on this study (upper panel) and sites where the leaf-level measurements were collected (lower panel). The eddy flux sites in blue in the upper panel were used to calibrate SOX hydraulic parameters (see the Material and Methods section in the main document).

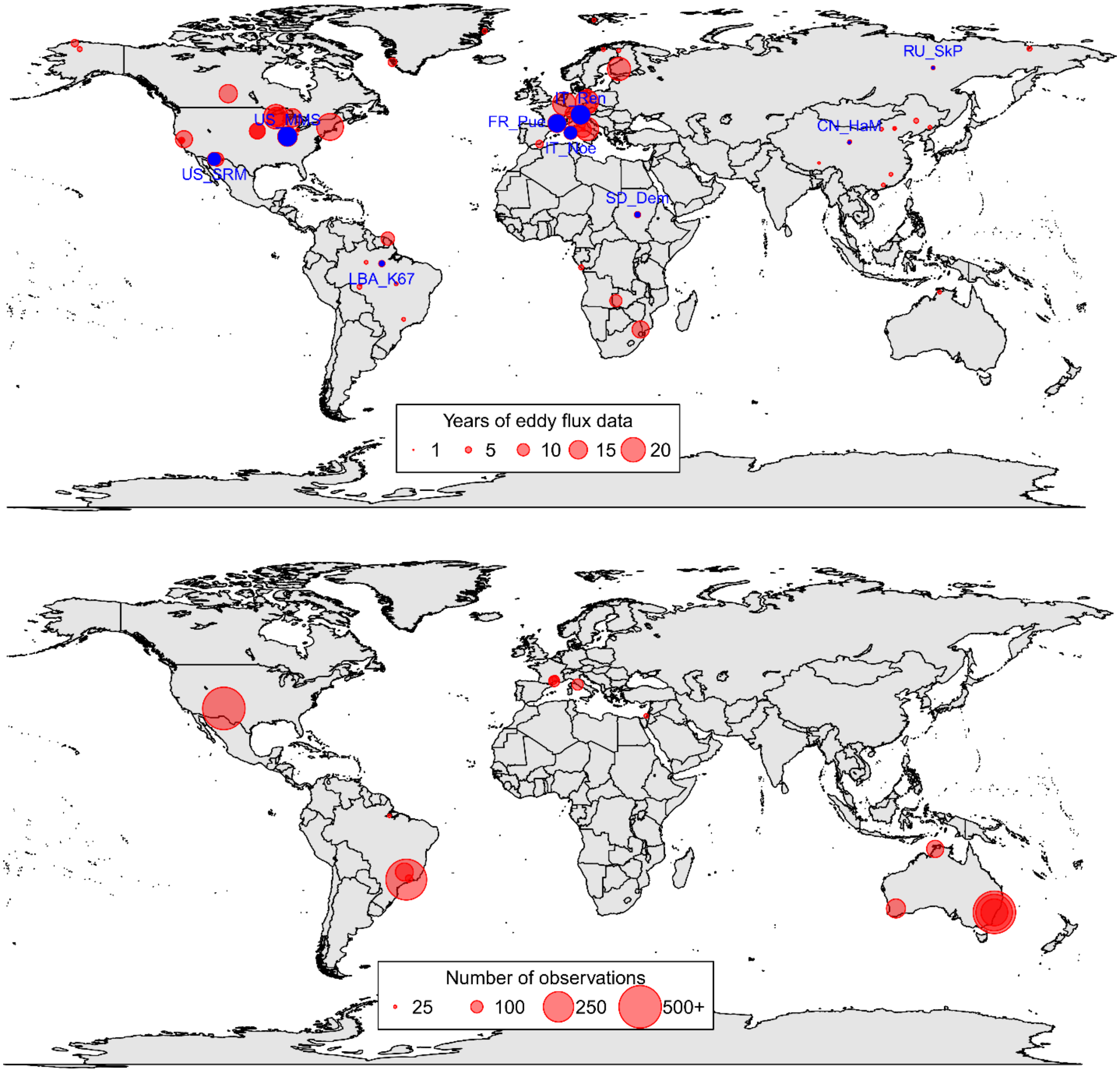

Fig. S3 Agreement between the numerical SOX model from Eller *et al.* (2018) with the analytical approximation presented in this study (Eqn 4-5). Each variable was changed individually while the other variables were held constant at their reference values, similarly to Figure 1 (D = 0.5 kPa, Ψ_{pd} = -0.5 MPa, I_{par} = 600 µmol m^{-2} s^{-1}, c_a = 36 Pa). The hydraulic parameters were set to Ψ_{50} = -2 MPa, a = 3, r_{pmin} =1 mmol^{-1} m^2 s MPa. The hydraulic parameters of the analytical model were fitted to the numerical model output. The V_{cmax25} was set to 100 µmol m-2 s and the rest of the photosynthetic parameters follow the BET-Tr parameterization from Harper *et al.* (2016). The *r* is the coefficient of correlation between models, and RMSE the root mean square error.

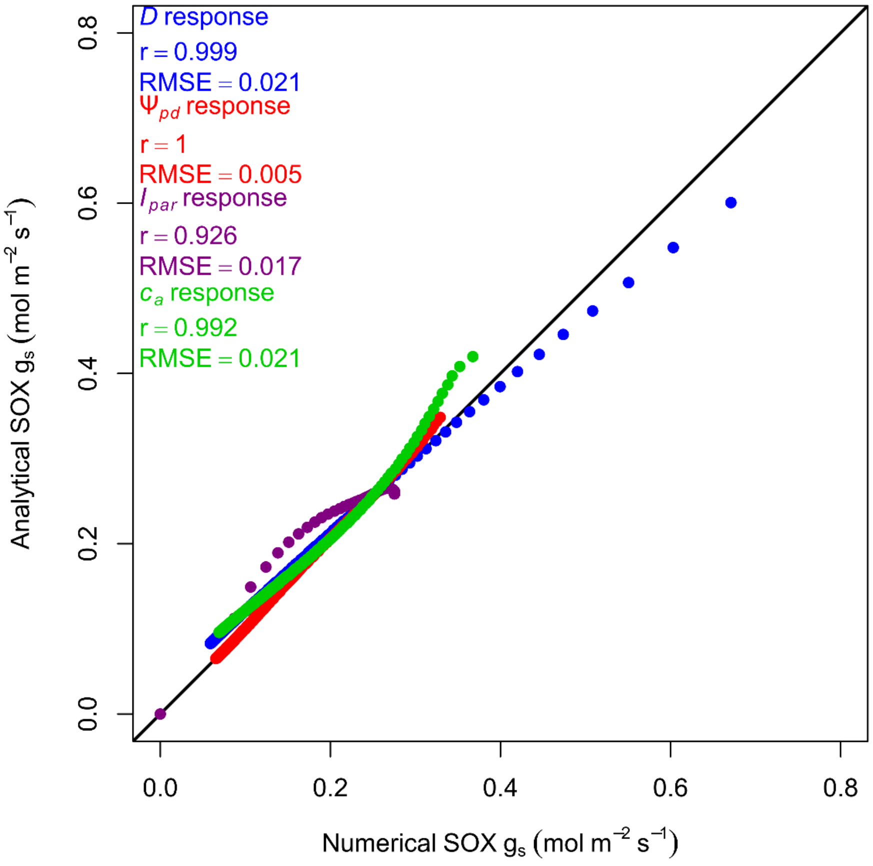

Fig. S4 Daily drought evolution during two months before the JULES predicts the lowest soil moisture for each of the calibration sites (see Table S2 and Fig. S3). The data in deciduous ecosystems was restricted to the growing season. The blue and red lines in the upper panel are the daily gross primary productivity (GPP) modelled by JULES and JULES-SOX, respectively. The grey points are eddy covariance derived GPP. The red line in the mid-panel is the JULES-SOX modelled minimum daily water potential (Ψ_{midday}) and the blue line is the β factor from JULES. The bottom panel shows the mean daily soil moisture at the top 1 m of soil expressed as a fraction of the soil moisture saturation point as modelled by JULES (blue) and JULES-SOX (red). The bars indicate the daily rainfall. The PFT abbreviations are: **(a)** BET-Tr (Broadleaf evergreen tropical tree), **(b)** BET-Te (Broadleaf evergreen temperate tree), **(c)** BDT (Broadleaf deciduous tree), **(d)** NET (Needleleaf evergreen tree), **(e)** NDT (Needleleaf deciduous tree), **(f)** C3 (C3 grasses), **(g)** C4 (C4 grasses), **(h)** ESh (Evergreen shrubs) and **(i)** DSh (Deciduous shrubs).

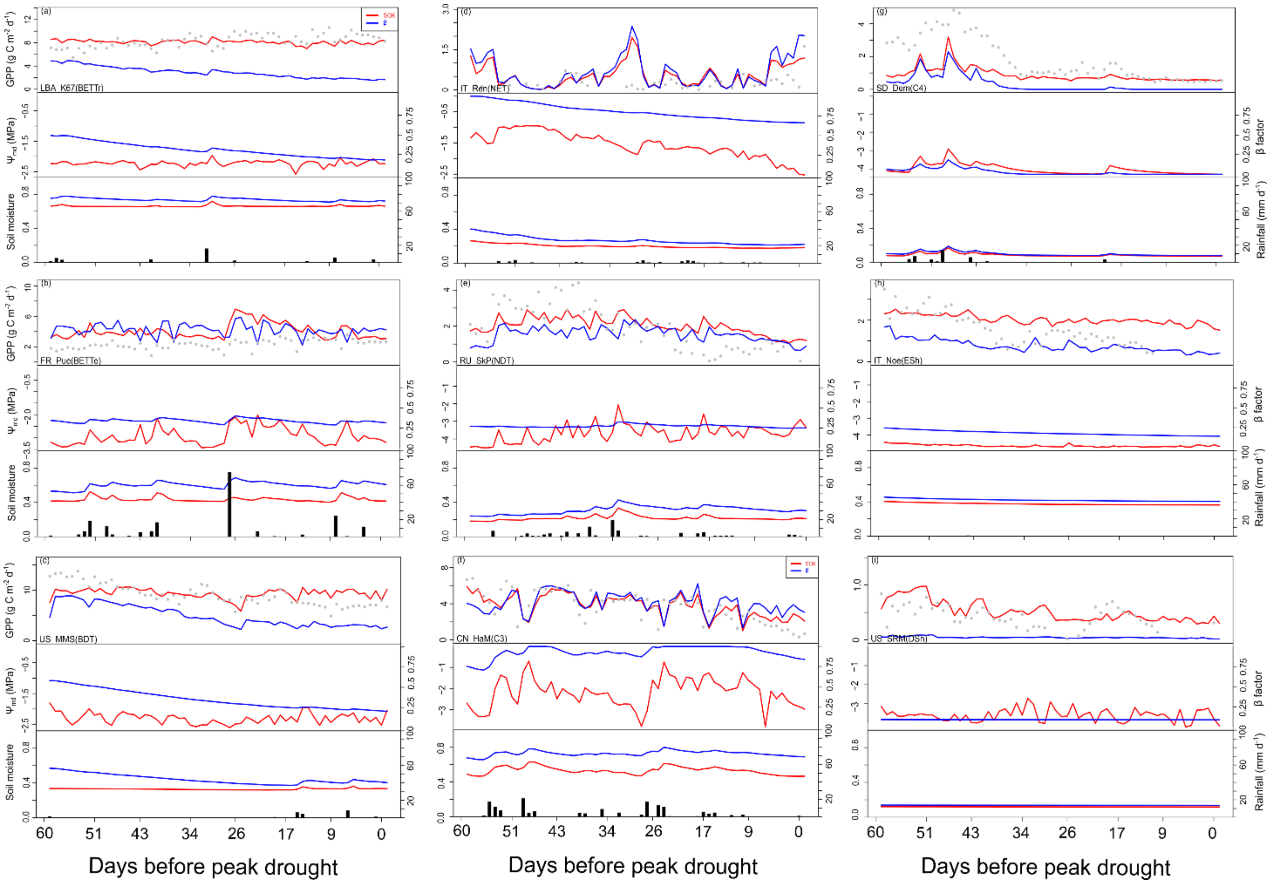

LBA_K67(BETTr)
FR_Pue(BETTe)
US_MMS(BDT)
IT_Ren(NET)
RU_SkP(NDT)
CN_HaM(C3)
SD_Dem(C4)
IT_Noe(ESh)
US_SRM(DSh)
GPP (g C m-2 d-1)
Ψmd (MPa)
Soil moisture
β factor
Rainfall (mm d-1)
SOX
β
Days before peak drought
Days before peak drought
Days before peak drought

Fig. S5 Monthly mean differences between evapotranspiration (ET) predicted by JULES-SOX from ET predicted by default JULES. at each eddy flux site used for calibrating the SOX hydraulic parameters (PFT; Table S2 and Fig. S3). The PFT abbreviations are: **(a)** BET-Tr (Broadleaf evergreen tropical tree), **(b)** BET-Te (Broadleaf evergreen temperate tree), **(c)** BDT (Broadleaf deciduous tree), **(d)** NET (Needleleaf evergreen tree), **(e)** NDT (Needleleaf deciduous tree), **(f)** C3 (C3 grasses), **(g)** C4 (C4 grasses), **(h)** ESh (Evergreen shrubs), and **(i)** DSh (Deciduous shrubs).

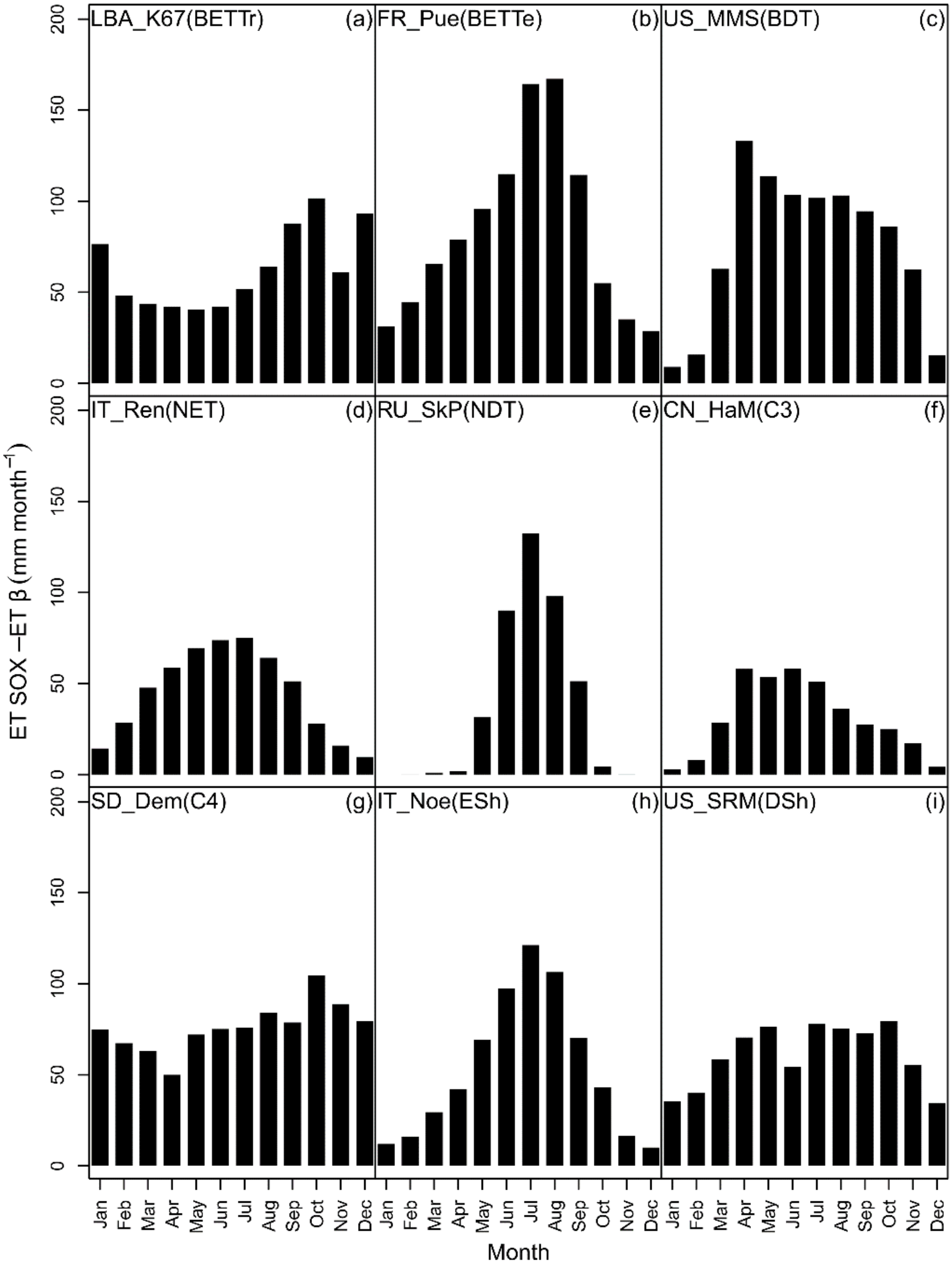
LBA_K67(BETTr)
(a)
FR_Pue(BETTe)
(b)
US_MMS(BDT)
(c)
IT_Ren(NET)
(d)
RU_SkP(NDT)
(e)
CN_HaM(C3)
(f)
SD_Dem(C4)
(g)
IT_Noe(ESh)
(h)
US_SRM(DSh)
(i)
ET SOX −ET β (mm month−1)
0
50
100
150
200
Jan
Feb
Mar
Apr
May
Jun
Jul
Aug
Sep
Oct
Nov
Dec
Month

Fig. S6 Normalized monthly mean evapotranspiration (ET) predicted by default JULES (blue line) and JULES-SOX (red line). The grey points are observations derived from eddy covariance latent heat measurements. The model fit to data is shown as the root mean squared errors (RMSE) and Nash-Sutcliffe (1970) model efficiency index (NSE). The normalization of the data was conducted to minimize the bias in the eddy covariance latent heat observations due to the lack of energy balance closure (Twine *et al.* 2000). We had no latent heat data for the LBA_K67 site. The PFT abbreviations are: **(a)** BET-Tr (Broadleaf evergreen tropical tree), **(b)** BET-Te (Broadleaf evergreen temperate tree), **(c)** BDT (Broadleaf deciduous tree), **(d)** NET (Needleleaf evergreen tree), **(e)** NDT (Needleleaf deciduous tree), **(f)** C3 (C3 grasses), **(g)** C4 (C4 grasses), **(h)** ESh (Evergreen shrubs), and **(i)** DSh (Deciduous shrubs).

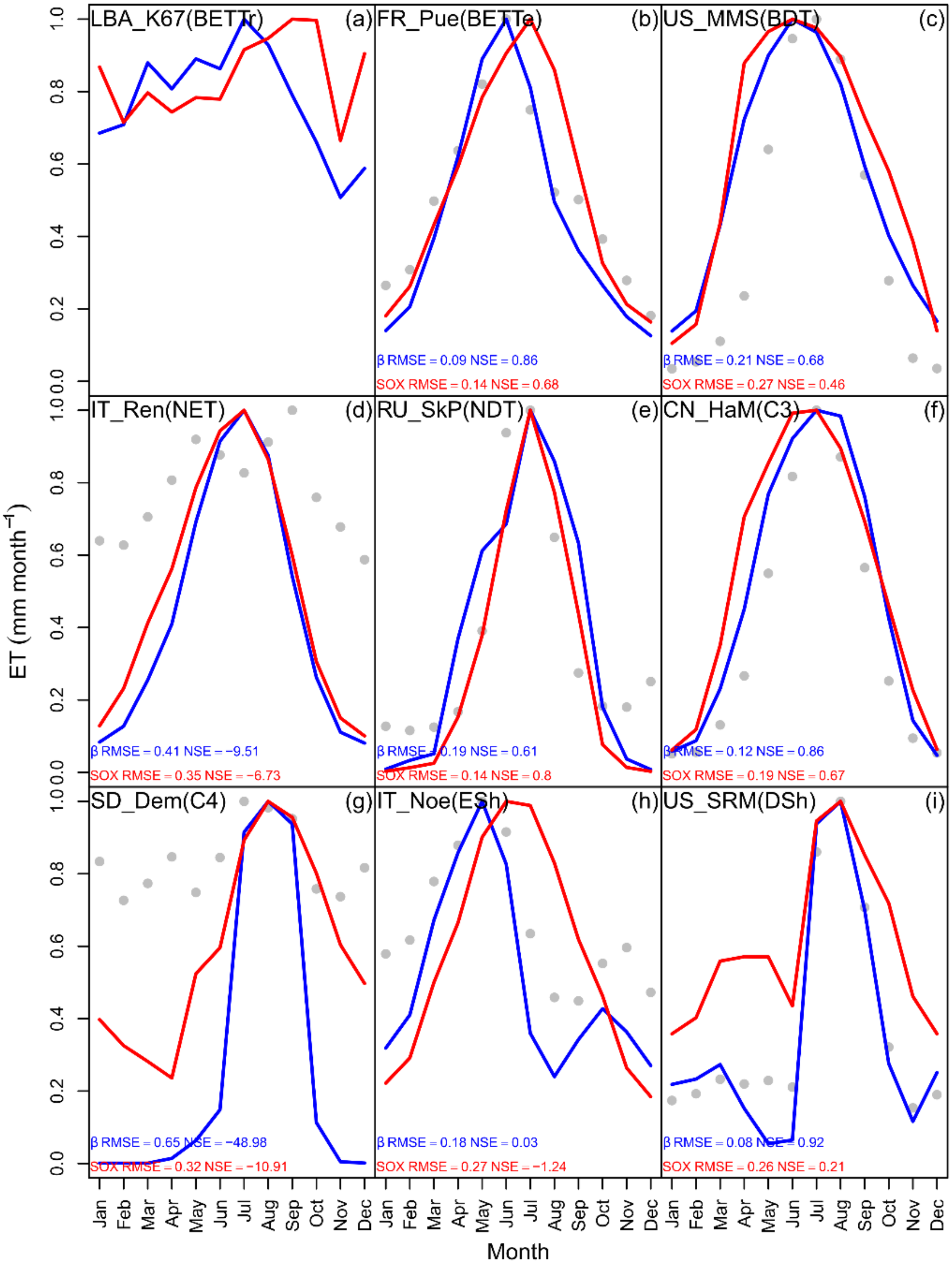

LBA_K67(BETTr)
(a)
FR_Pue(BETTe)
(b)
β RMSE = 0.09 NSE = 0.86
SOX RMSE = 0.14 NSE = 0.68
US_MMS(BDT)
(c)
β RMSE = 0.21 NSE = 0.68
SOX RMSE = 0.27 NSE = 0.46
IT_Ren(NET)
(d)
β RMSE = 0.41 NSE = −9.51
SOX RMSE = 0.35 NSE = −6.73
RU_SkP(NDT)
(e)
β RMSE = 0.19 NSE = 0.61
SOX RMSE = 0.14 NSE = 0.8
CN_HaM(C3)
(f)
β RMSE = 0.12 NSE = 0.86
SOX RMSE = 0.19 NSE = 0.67
SD_Dem(C4)
(g)
β RMSE = 0.65 NSE = −48.98
SOX RMSE = 0.32 NSE = −10.91
IT_Noe(ESh)
(h)
β RMSE = 0.18 NSE = 0.03
SOX RMSE = 0.27 NSE = −1.24
US_SRM(DSh)
(i)
β RMSE = 0.08 NSE = 0.92
SOX RMSE = 0.26 NSE = 0.21
ET (mm month^{-1})
Month
Jan Feb Mar Apr May Jun Jul Aug Sep Oct Nov Dec

Fig. S7 Monthly mean soil moisture normalized in function of the highest observed value at the top 0.1 m soil layer as predicted by default JULES (blue line) and JULES-SOX (red line). The grey points are normalized soil moisture observations measured at 5 to 15 cm depth. The model fit to data is shown as the root mean squared errors (RMSE) and Nash-Sutcliffe (1970) model efficiency index (NSE). The PFT abbreviations are: **(a)** BET-Tr (Broadleaf evergreen tropical tree), **(b)** BET-Te (Broadleaf evergreen temperate tree), **(c)** BDT (Broadleaf deciduous tree), **(d)** NET (Needleleaf evergreen tree), **(e)** NDT (Needleleaf deciduous tree), **(f)** C3 (C3 grasses), **(g)** C4 (C4 grasses), **(h)** ESh (Evergreen shrubs), and **(i)** DSh (Deciduous shrubs).

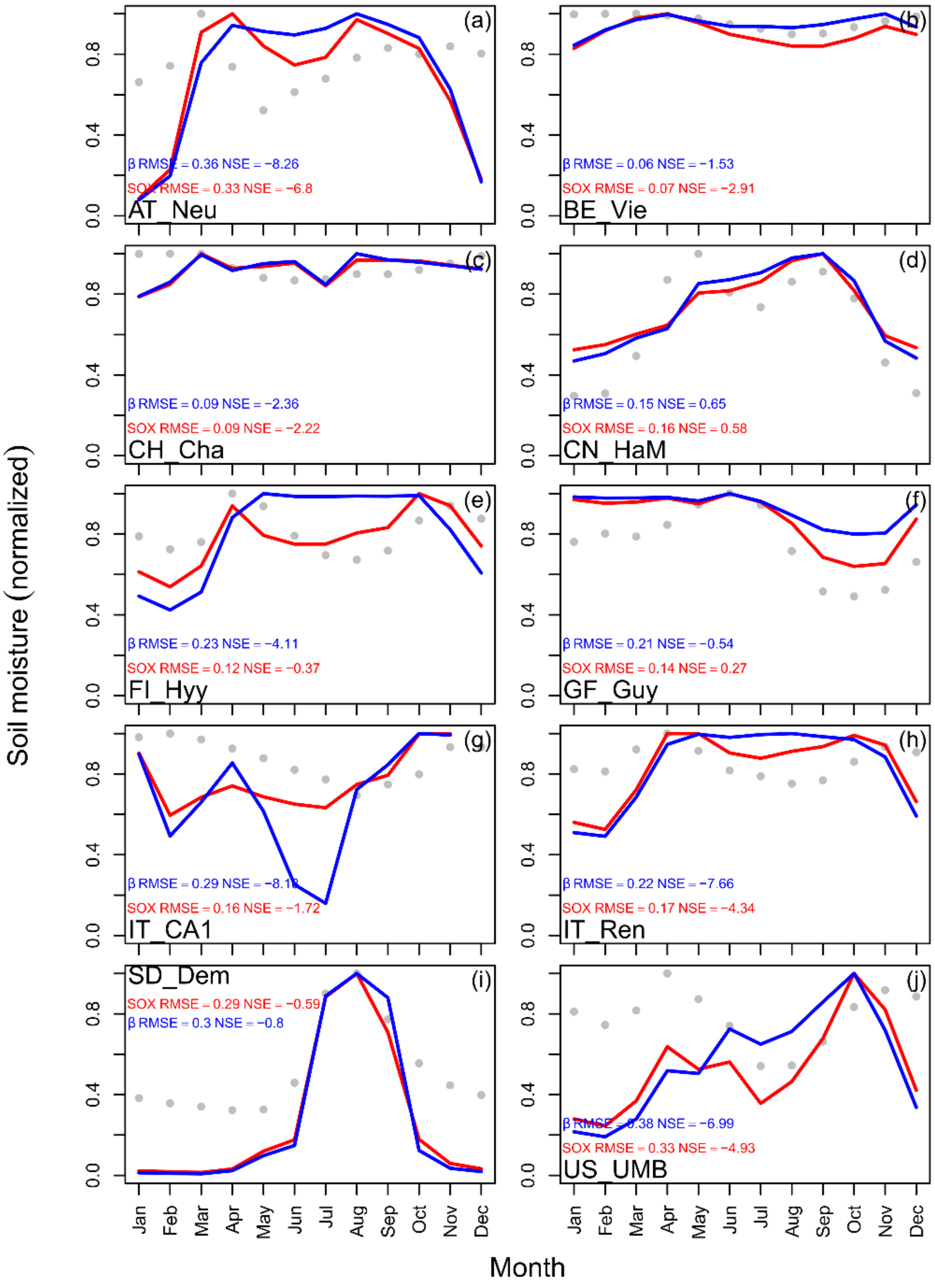

Soil moisture (normalized)
Month
(a)
β RMSE = 0.36 NSE = −8.26
SOX RMSE = 0.33 NSE = −6.8
AT_Neu
(b)
β RMSE = 0.06 NSE = −1.53
SOX RMSE = 0.07 NSE = −2.91
BE_Vie
(c)
β RMSE = 0.09 NSE = −2.36
SOX RMSE = 0.09 NSE = −2.22
CH_Cha
(d)
β RMSE = 0.15 NSE = 0.65
SOX RMSE = 0.16 NSE = 0.58
CN_HaM
(e)
β RMSE = 0.23 NSE = −4.11
SOX RMSE = 0.12 NSE = −0.37
FI_Hyy
(f)
β RMSE = 0.21 NSE = −0.54
SOX RMSE = 0.14 NSE = 0.27
GF_Guy
(g)
β RMSE = 0.29 NSE = −8.1
SOX RMSE = 0.16 NSE = −1.72
IT_CA1
(h)
β RMSE = 0.22 NSE = −7.66
SOX RMSE = 0.17 NSE = −4.34
IT_Ren
(i)
SD_Dem
SOX RMSE = 0.29 NSE = −0.59
β RMSE = 0.3 NSE = −0.8
(j)
β RMSE = 0.38 NSE = −6.99
SOX RMSE = 0.33 NSE = −4.93
US_UMB
0.0
0.4
0.8
Jan
Feb
Mar
Apr
May
Jun
Jul
Aug
Sep
Oct
Nov
Dec

Table S1 Species, sites and sources for the data used on the SOX leaf-level evaluation.

PFT	Species	Lat	Lon	Source
Broadleaf evergreen tropical tree (BET-Tr)	*Acacia mimula*	-12.4	130.9	Thomas & Eamus (2002)
	Eucalyptus tetrodonta	-12.4	130.9	Thomas & Eamus (2002)
	Eremanthus erythropappus	-22.7	-45.6	Eller *et al*. (2016)
	Myrsine umbellata	-22.7	-45.6	Eller *et al*. (2016)
	Manilkara Bidentata	-1.43	-51.2	Rowland *et al*. (2015)
	Licaria armeniaca	-1.43	-51.2	Rowland *et al.* (2015)
	Mouriri duckeana	-1.43	-51.2	Rowland *et al*. (2015)
Broadleaf evergreen temperate tree (BET-Te)	*Quercus ilex*	43.48; 42.3	3.75; 11.6	Martin-St. Paul *et al.* (2012); De Angelis & Scarascia-Mugnozza (1998)
	Angophora bakeri	-33.6	150.6	Zeppel *et al*. (2008)
	Eucalyptus parramattensis	-33.6	150.6	Zeppel *et al*. (2008)
	Eucalyptus saligna	-33.6	150.7	Barton *et al*. (2012); Heroult *et al*. (2013)
	Eucalyptus tereticornis	-33.6	150.7	Gimeno *et al*. (2016)
	Eucalyptus dunnii	-33.6	150.7	Heroult *et al*. (2013)
	Eucalyptus melliodora	-33.6	150.7	Heroult *et al*. (2013)
	Eucalyptus cladocalyx	-33.6	150.7	Heroult *et al*. (2013)
	Eucalyptus capillosa	-32.3	117.8	Mitchell *et al*. (2009)
	Eucalyptus salmonophloiia	-32.3	117.8	Mitchell *et al*. (2009)
	Citrus Limon	31.9	34.5	Wagner Y & Klein T, *unpublished*
Broadleaf deciduous tree (BDT)	*Alphitonia excelsa*	-12.4	130.9	Thomas & Eamus (2002)
	Cochlospermum fraseri	-12.4	130.9	Thomas & Eamus (2002)
	Planchonia careya	-12.4	130.9	Thomas & Eamus (2002)
Needleleaf	*Juniperus monosperma*	34.4	-106.5	Limousin *et al*. (2013)

evergreen tree (NET)	*Pinus edulis*	34.4	-106.5	Limousin *et al*. (2013)
Evergreen shrub (ESh)	*Phillyrea angustifolia*	42.3	11.6	De Angelis & Scarascia-Mugnozza (1998)
	Eremanthus seidelii	-20.1	-46.2	Teodoro (2014)
	Campomanesia pubescens	-20.1	-46.2	Teodoro (2014)
	Pleroma hospita	-22.6	-44.1	Matos IS & Rosado BHP, *unpublished*
	Chionolaena capitata	-22.6	-44.1	Matos IS & Rosado BHP, *unpublished*
Deciduous shrub (DSh)	*Vernonia warmingiana*	-20.1	-46.2	Teodoro (2014)
	Mimosa clausenii	-20.1	-46.2	Teodoro (2014)

Table S2 Sites used for the ecosystem-level evaluation of JULES-SOX

Site code	Lat	Lon	Category	PFT cover	Source
AT_Neu	47.11	11.31	GRA	80% C3, 20% Soil	FLUXNET – DOI: 10.18140/FLX/1440121
AU_Fog	-12.54	131.31	WET	75% C4, 20% BDT, 5% Soil	FLUXNET – DOI: 10.18140/FLX/1440124
BE_Vie	50.30	5.99	MF	35% BDT, 35% NET, 20% C3, 10% Urban	FLUXNET – DOI: 10.18140/FLX/1440130
CA_Oas	53.63	-106.19	MF	35% BDT, 35% NET, 20% C3, 10% Urban	FLUXNET – DOI: 10.18140/FLX/1440043
CG_Tch	-4.29	11.66	SAV	50% BDT, 25% ESh, 15%	FLUXNET – DOI:

				C4, 10% Urban	10.18140/FLX/144 0142
CH_Cha	47.21	8.41	GRA	80% C3, 20% Soil	FLUXNET – DOI: 10.18140/FLX/144 0131
CN_Cha	42.40	128.08	MF	35% BDT, 35% NET, 20% C3, 10% Urban	FLUXNET – DOI: 10.18140/FLX/144 0137
CN_Cng	44.59	123.51	GRA	70% C3, 15% C4, 15% Soil	FLUXNET – DOI: 10.18140/FLX/144 0209
CN_Dan	30.49	91.06	GRA	70% C3, 15% C4, 15% Soil	FLUXNET – DOI: 10.18140/FLX/144 0138
CN-Din	23.17	112.53	EBF-Tr	100% BET-Tr	FLUXNET – DOI: 10.18140/FLX/144 0139
CN-Du2	42.04	116.28	GRA	70% C3, 15% C4, 15% Soil	FLUXNET – DOI: 10.18140/FLX/144 0140
CN-Du3	42.05	116.28	GRA	70% C3, 15% C4, 15% Soil	FLUXNET – DOI: 10.18140/FLX/144 0210
CN_Ha2	37.61	101.32	WET	80% C3, 20% Water	FLUXNET – DOI: 10.18140/FLX/144 0211
CN_Ham	**37.37**	**101.18**	**GRA**	**80% C3, 20% Soil**	**FLUXNET – DOI: 10.18140/FLX/144 0190**
CN_Qia	26.74	115.05	ENF	100% NET	FLUXNET – DOI: 10.18140/FLX/144

					0141
CZ_wet	49.02	14.77	WET	80% C3, 20% Water	FLUXNET – DOI: 10.18140/FLX/1440145
DE_Akm	53.86	13.68	WET	80% C3, 20% Water	FLUXNET – DOI: 10.18140/FLX/1440213
DE_SfN	47.81	11.33	WET	80% C3, 20% Water	FLUXNET – DOI: 10.18140/FLX/1440219
DE_Spw	51.89	14.03	WET	80% C3, 20% Water	FLUXNET – DOI: 10.18140/FLX/1440220
DE_Tha	50.96	13.56	ENF	100% NET	FLUXNET – DOI: 10.18140/FLX/1440152
DE_Zrk	53.87	12.89	WET	80% C3, 20% Water	FLUXNET – DOI: 10.18140/FLX/1440221
DK_NuF	64.13	-51.38	WET	80% C3, 20% Water	FLUXNET – DOI: 10.18140/FLX/1440222
DK_ZaF	74.48	-20.55	WET	80% C3, 20% Water	FLUXNET – DOI: 10.18140/FLX/1440223
ES_Amo	56.83	-2.25	SHR	55% C4, 35% Dsh, 10% Urban	FLUXNET – DOI: 10.18140/FLX/1440156
FI_Hyy	61.85	24.29	ENF	100% NET	FLUXNET – DOI: 10.18140/FLX/1440158

FI_Lom	67.99	24.21	WET	80% C3, 20% Water	FLUXNET – DOI: 10.18140/FLX/1440228
FR_Pue	**43.74**	**3.59**	**EBF-Te**	**100% BET-Te**	**FLUXNET – DOI: 10.18140/FLX/1440164**
GF_Guy	5.28	-52.92	EBF-Tr	100% BET-Tr	FLUXNET – DOI: 10.18140/FLX/1440165
IT_Col	41.85	13.59	DBF	100% BDT	FLUXNET – DOI: 10.18140/FLX/1440167
IT_Cp2	41.85	13.59	EBF-Te	100% BET-Te	FLUXNET – DOI: 10.18140/FLX/1440233
IT_Cpz	41.70	12.38	EBF-Te	100% BET-Te	FLUXNET – DOI: 10.18140/FLX/1440168
IT_Noe	**40.61**	**8.15**	**SHR**	**60% ESh, 10% C3, 10% C4, 20% Soil**	**FLUXNET – DOI: 10.18140/FLX/1440171**
IT_Ren	**46.59**	**11.43**	**ENF**	**100% NET**	**FLUXNET – DOI: 10.18140/FLX/1440173**
IT_SRo	43.73	10.28	ENF	100% NET	FLUXNET – DOI: 10.18140/FLX/1440176
NO_Adv	78.18	15.92	WET	80% C3, 20% Water	FLUXNET – DOI: 10.18140/FLX/1440241
RU_Che	68.61	161.34	WET	80% C3, 20% Soil	FLUXNET – DOI:

					10.18140/FLX/1440181
RU_SkP	**62.25**	**129.17**	**DNF**	**80% NDT, 10% BDT, 10% NET**	**FLUXNET – DOI: 10.18140/FLX/1440243**
SD_Dem	13.28	30.48	GRA	85% C4, 15% Soil	FLUXNET – DOI: 10.18140/FLX/1440186
SE_St1	68.35	19.05	WET	80% C3, 20% Water	FLUXNET – DOI: 10.18140/FLX/1440187
US_Atq	70.47	-157.41	WET	80% C3, 20% Water	FLUXNET – DOI: 10.18140/FLX/1440067
US_Ha1	42.54	-72.17	DBF	100% BDT	FLUXNET – DOI: 10.18140/FLX/1440071
US_Ivo	68.48	-155.75	WET	80% C3, 20% Water	FLUXNET – DOI: 10.18140/FLX/1440073
US_Los	46.08	-89.98	WET	80% C3, 20% Water	FLUXNET – DOI: 10.18140/FLX/1440076
US_MMS	**39.32**	**-86.41**	**DBF**	**100% BDT**	**FLUXNET – DOI: 10.18140/FLX/1440083**
US_Myb	38.05	-121.76	WET	80% C3, 20% Water	FLUXNET – DOI: 10.18140/FLX/1440105
US_Ne1	41.16	-96.48	CRO	100% C4	FLUXNET – DOI: 10.18140/FLX/144

					0084
US_Ne2	41.16	-96.47	CRO	50% C3, 50% C4	FLUXNET – DOI: 10.18140/FLX/1440085
US_Ne3	41.18	-96.44	CRO	50% C3, 50% C4	FLUXNET – DOI: 10.18140/FLX/1440086
US_ORv	40.02	-83.02	WET	80% C3, 20% Water	FLUXNET – DOI: 10.18140/FLX/1440102
US_PFa	45.94	-90.27	BDF	70% BDT, 30% NET	FLUXNET – DOI: 10.18140/FLX/1440089
US_SRG	31.79	-110.83	GRA	85% C4, 15% Soil	FLUXNET – DOI: 10.18140/FLX/1440114
US_SRM	**31.82**	**-110.87**	**SHR**	**55% C4, 35% DSh, 10% Urban**	**FLUXNET – DOI: 10.18140/FLX/1440090**
US_Ton	38.43	-120.96	SAV	30% BDT, 30% C3, 30% DSh, 10% Soil	FLUXNET – DOI: 10.18140/FLX/1440092
US_Tw1	38.11	-121.65	WET	80% C3, 20% Water	FLUXNET – DOI: 10.18140/FLX/1440108
US_Tw4	38.10	-121.64	WET	80% C3, 20% Water	FLUXNET – DOI: 10.18140/FLX/1440111
US_UMB	45.56	-84.71	DBF	100% BDT	FLUXNET – DOI: 10.18140/FLX/1440093

US_WCr	45.80	-90.08	DBF	100% BDT	FLUXNET – DOI: 10.18140/FLX/1440095
US_Whs	31.74	-110.05	SHR	40% DSh, 40% ESh, 10% C4, 10% Urban	FLUXNET – DOI: 10.18140/FLX/1440097
US_Wkg	31.74	-109.94	GRA	85% C4, 15% Soil	FLUXNET – DOI: 10.18140/FLX/1440096
US_WPT	41.46	-82.99	WET	80% C3, 20% Water	FLUXNET – DOI: 10.18140/FLX/1440116
ZA_Kru	-25.02	31.49	SAV	75% C4, 20% BDT, 5% Soil	FLUXNET – DOI: 10.18140/FLX/1440188
ZM_Mon	-15.44	23.25	DBF	100% BDT	FLUXNET – DOI: 10.18140/FLX/1440189
LBA_BAN	-9.81	-50.13	WET	50% BDT, 25% ESh, 15% C4, 10% Urban	LBA
LBA_FNS	-10.76	-62.36	GRA	85% C4, 15% Soil	LBA
LBA_K34	-2.60	-60.12	EBF-Tr	100% BET-Tr	LBA
LBA_K67	**-2.85**	**-54.96**	**EBF-Tr**	**100% BET-Tr**	**LBA**
LBA_K77	-3.01	-54.88	GRA	85% C4, 15% Soil	LBA
LBA_K83	-3.05	-54.93	EBF-Tr	100% BET-Tr	LBA
LBA_PDG	-21.61	-47.61	SAV	50% BDT, 25% ESh, 15% C4, 10% Urban	LBA
LBA_RJA	-10.07	-61.93	EBF-Tr	100% BET-Tr	LBA

Note: Urban, Water and Soil are JULES non-vegetation surface cover types, see details in Best et al. (2011). The rest of the abbreviations are as follows: Cropland (CRO), deciduous broadleaf

forests (DBF), deciduous needleleaf forests (DNF), temperate evergreen broadleaf forests (EBF-Te), tropical evergreen broadleaf forests (EBF-Tr), evergreen needleleaf forest (ENF), grassland (GRA), mixed forest (MF), savannah (SAV), shrubland (SHR), and wetlands (WET).

Notes S1 The mean (Ψ_m; MPa) of the canopy water potential gradient at predawn (Ψ_{pd}; MPa) and the canopy water potential (Ψ_c; MPa) to compute K with the xylem vulnerability (Eqn 2 from main text) is defined as:

$$\Psi_m = \frac{(\Psi_{pd}+\Psi_c)}{2} \quad \text{(S1.1a)}$$

hence:

$$\Psi_c = 2\Psi_m - \Psi_{pd} \quad \text{(S1.1b)}$$

where Ψ_{pd} is computed assuming no nighttime transpiration, and is therefore equal to the mean soil water potential in the root zone (Ψ_r; MPa) minus the canopy height induced Ψ gradient:

$$\Psi_{pd} = \Psi_r - hg\rho 10^{-6} \quad \text{(S1.2)}$$

where h is the canopy height (m), g is the acceleration due to gravity (9.8 m s^{-2}), ρ is the water density (997 kg m^{-3} at 25° C) and the 10^{-6} converts Pa (kg m^{-1} s^{-2}) to MPa. Eller et al (2018) shows that the use of $K(\Psi_m)$ approximates the results of the Kirchhoff integral transform employed by Sperry & Love (2015), that is:

$$K(\Psi_m) \approx \frac{\int_{\Psi_c}^{\Psi_{pd}} K(\Psi)d\Psi}{(\Psi_{pd}-\Psi_c)} \quad \text{(S1.3)}$$

The g_s value that maximises equation 1 from the main text is found at:

$$\frac{\partial AK}{\partial g_s} = 0 \quad \text{(S1.4a)}$$

or

$$\frac{1}{A}\frac{\partial A}{\partial g_s} = \frac{-1}{K}\frac{\partial K}{\partial g_s} \quad \text{(S1.4b)}$$

The g_s value that satisfies equation S1.4 was found numerically in Eller et al (2018). However, a computationally efficient analytical solution is preferable to numerical approaches in large-scale vegetation modelling. Therefore, in this study we develop an analytical approximation for the optimal SOX g_s using the partial derivatives of A with respect to c_i and K with respect to Ψ_m. In this approach, the increased costs of stomatal aperture under low Ψ_{pd} and high leaf-to-air vapour

pressure deficit (D; mol mol^{-1}) are synthesized in the parameter ξ (Eqn 5 from main text; Fig. S1a). The parameter ξ is derived from the rate of change in K with respect to g_s ($\partial K/\partial g_s$), which can be written as:

$$\frac{\partial K}{\partial g_s} = \frac{\partial K}{\partial \Psi_m}\frac{\partial \Psi_m}{\partial g_s} \tag{S1.5}$$

The canopy transpiration (E; mol H_2O m^{-2} s^{-1}) in steady state is equal to the water flux inside the plant xylem which can be described using an Ohm's law analogy:

$$E = \frac{(\Psi_{pd} - \Psi_c)}{r_p} \tag{S1.6a}$$

$$\Psi_c = \Psi_{pd} - r_p E \tag{S1.6b}$$

where r_p is the plant hydraulic resistance (mol^{-1} H_2O m^2 s MPa). Substituting equation S1.1b into equation S1.6b gives:

$$2\Psi_m - \Psi_{pd} = \Psi_{pd} - r_p E \tag{S1.7a}$$

$$\Psi_m = \Psi_{pd} - \frac{r_p E}{2} \tag{S1.7b}$$

We can use Fick's law of diffusion to write a second expression for E as a function of g_s and D:

$$E = 1.6 g_s D \tag{S1.8}$$

where 1.6 is the ratio between the molecular H_2O and CO_2 diffusivity in the atmosphere. Substituting equation S1.8 into equation S1.7b gives:

$$\Psi_m = \Psi_{pd} - \frac{r_p 1.6 g_s D}{2} \tag{S1.9}$$

which can be differentiated with respect to g_s into:

$$\frac{\partial \Psi_m}{\partial g_s} = \frac{-r_p 1.6 D}{2} \tag{S1.10}$$

Equation S1.10 can then be substituted into equation S1.5:

$$\frac{-1}{K}\frac{\partial K}{\partial g_s} = \frac{1}{K}\frac{\partial K}{\partial \Psi_m}\frac{r_p 1.6 D}{2} \tag{S1.11}$$

Substituting equation S1.11 into equation S1.4b gives:

$$\frac{1}{A}\frac{\partial A}{\partial g_s} = \frac{1}{K}\frac{\partial K}{\partial \Psi_m}\frac{r_p 1.6 D}{2} \tag{S1.12a}$$

or

$$\frac{1}{A}\frac{\partial A}{\partial g_s} = \frac{1}{\xi} \tag{S1.12b}$$

Where ξ is defined as equation 5 in the main text.

Solving for the SOX optimum g_s that satisfies equation S1.4b also requires expressing the rate of change in A with respect to g_s ($\partial A/\partial g_s$) from equation S1.12b as a function of g_s, which can be done as:

$$\frac{\partial A}{\partial g_s} = \frac{\partial A}{\partial c_i}\frac{\partial c_i}{\partial g_s} \tag{S1.13}$$

and from Fick's law we can write c_i as:

$$c_i = c_a - \frac{A}{g_s} \tag{S1.14a}$$

$$\frac{\partial c_i}{\partial g_s} = \frac{1}{g_s}\cdot\left(\frac{A}{g_s} - \frac{\partial A}{\partial g_s}\right) \tag{S1.14b}$$

Replacing equation S1.14b into equation S1.13 gives:

$$\frac{\partial A}{\partial g_s} = \frac{1}{g_s}\left(\frac{A}{g_s} - \frac{\partial A}{\partial g_s}\right)\frac{\partial A}{\partial c_i} \tag{S1.15a}$$

$$\frac{\partial A}{\partial g_s} = \frac{A}{g_s(g_s+\partial A/\partial c_i)}\frac{\partial A}{\partial c_i} \tag{S1.15b}$$

we can now replace equation S1.15b into equation S1.12b:

$$\frac{1}{A}\frac{A}{g_s(g_s+\partial A/\partial c_i)}\frac{\partial A}{\partial c_i} = \frac{1}{\xi} \tag{S1.16}$$

equation S1.16 can then be rearranged as a quadratic equation:

$$g_s\left(g_s + \frac{\partial A}{\partial c_i}\right) = \xi\,\frac{\partial A}{\partial c_i} \tag{S1.17a}$$

$$g_s^2 + g_s\frac{\partial A}{\partial c_i} - \xi\,\frac{\partial A}{\partial c_i} = 0 \tag{S1.17b}$$

The SOX optimum g_s defined in equation 4 from the main text is the physically meaningful solution of equation S1.17b, which in this case corresponds to its negative root.

Notes S2 The derivative $\partial A/\partial c_i$ is computed numerically by solving the Collatz et al (1992, 1991) C_3 and C_4 photosynthesis models at c_a and at the co-limitation c_i ($c_{i,col}$), which is the point where increasing c_i will no longer increase A, as carbon assimilation is being limited by light or transport of photoassimilates.

$$\frac{\partial A}{\partial c_i} = \frac{[A(c_a) - A(c_{i,col})]}{(c_a - c_{i,col})} \tag{S2. 1}$$

The $\partial A/\partial c_i$ gradient calculated with equation S2.1 can adequately reproduce stomatal closure at high c_a and low light, which results in lower $\partial A/\partial c_i$ (Fig. S1b). When the $\partial A/\partial c_i$ gradient goes to 0, such as in low-light conditions, g_s is set to the g_s at $c_{i,col}$, as there is no benefit for the plant, in terms of carbon uptake, to surpass the g_s at $c_{i,col}$:

$$\frac{\partial A}{\partial c_i} = 0 \Rightarrow g_s = \frac{A(c_{i,col})P_a}{(c_a - c_{i,col})} \tag{S2.2}$$

The $c_{i,col}$, which corresponds to the c_i where the leaf gross photosynthesis rates (W; mol CO_2 m^{-2} s^{-1}) is no longer limited by c_i, can be found analytically rearranging the Collatz *et al*. (1992, 1991) photosynthesis model. Collatz *et al*. (1992, 1991) computes the rates of Rubisco limited carbon assimilation (W_c), light-limited carbon assimilation (W_l) and transport limited carbon assimilation (W_e) as:

$$W_c = \begin{cases} V_{cmax}\left(\frac{c_i - \Gamma}{c_i + K_c(1 + O_a/K_o)}\right) for C_3 \\ V_{cmax} \qquad\qquad\qquad\qquad for C_4 \end{cases} \tag{S2.3a}$$

$$W_l = \begin{cases} \alpha(1-\omega)I_{PAR}\left(\frac{c_i - \Gamma}{c_i + 2\Gamma}\right) for C_3 \\ \alpha(1-\omega)I_{PAR} \qquad\qquad for C_4 \end{cases} \tag{S2.3b}$$

$$W_e = \begin{cases} kV_{cmax} \quad for C_3 \\ kV_{cmax}\frac{c_i}{P_a} for C_4 \end{cases} \tag{S2.3c}$$

where V_{cmax} is the Rubisco maximum carboxylation rate (mol CO_2 m^{-2} s^{-1}), c_i is leaf internal CO_2 partial pressure (Pa), Γ is the photo-compensation point (Pa), O_a is the oxygen partial pressure in the atmosphere,(Pa), K_c and K_o are Rubisco carboxylation and oxygenation Michaelis-Menten constants (Pa), α is the photosynthesis quantum efficiency (mol CO_2 mol^{-1} I_{PAR}), ω is the leaf scattering coefficient for I_{PAR}, and I_{PAR} is the incident photosynthetic active radiation (mol photons m^{-2} s^{-1}), P_a is the surface atmospheric pressure (Pa), and k is a constant equal to 0.5 for C_3 plants and 2 x 10^{-4} for C_4 plants.

The $c_{i,col}$ can be found for C_3 plants at the value where the Rubisco-limited gross photosynthesis rate (W_c; mol CO_2 m^{-2} s^{-1}), which is strongly dependent on c_i (Eqn S2.3a), is equal to the co-limited W (W_{col}) of the W rates that do not depend strongly on c_i, that is, W_e and W_l. For simplicity, we ignored the weak dependency of W_l on c_i, simplifying equation S2.3b for C_3 plants to:

$$W_l = \alpha(1-\omega)I_{PAR} \tag{S2.4}$$

We can now find $c_{i,col}$ setting equation S2.3a equal to the co-limited W (W_{col}) between equation S2.4 and the equation S2.3c, which is given by the smallest root of the quadratic equation:

$$\beta W_{col}^2 - W_{col}(W_e + W_l) + W_e W_l = 0 \tag{S2.5}$$

Setting the root of equation S2.5 equal to equation S2.3a gives:

$$V_{cmax}\left(\frac{c_i-\Gamma}{c_i+K_c(1+O_a/K_o)}\right)=W_{col} \tag{S2.6a}$$

which can be rearranged into:

$$c_{i,col}=\frac{(-V_{cmax}\Gamma-K_c(1+O_a/K_o)W_{col})}{(W_{col}-V_{cmax})} \tag{S2.6b}$$

The same principle is applied to C_4 plants, but as the c_i dependency for C_4 plants is present on W_e, the quadratic to compute W_{col} becomes:

$$\beta W_{col}^2-W_{col}(W_c+W_l)+W_cW_l=0 \tag{S2.7}$$

Setting the root of equation S2.7 equal to equation S3.1c for C_4 plants gives:

$$kV_{cmax}\frac{c_i}{P_a}=W_{col} \tag{S2.8a}$$

which can be rearranged into:

$$c_{i,col}=\frac{W_{col}P_a}{kV_{cmax}} \tag{S2.8b}$$

The $\partial K/\Psi_m 1/K$ is computed as the linear gradient between K (Ψ_{pd}) and K ((Ψ_{pd}+Ψ_{50})/2):

$$\frac{\partial K}{\partial \Psi_m}\frac{1}{K}=\frac{K(\Psi_{pd})-K((\Psi_{pd}+\Psi_{50})/2)}{\Psi_{pd}-(\Psi_{pd}+\Psi_{50})/2}\frac{1}{K(\Psi_{pd})} \tag{S2.9}$$

We use the Ψ_{50} in equation S2.9 because it represents the steepest point of the vulnerability function $K(\Psi_m)$, producing a $\partial K/\partial\Psi_m 1/K > 0$ even in the "flat" portion of the vulnerability function. The linear assumption of equation S2.9 allows the stomata to respond dynamically to Ψ_m even during low water stress conditions. This avoids the problem raised by Buckley (2017) of similar stomatal optimization models (Wolf *et al.*, 2016; Sperry *et al.*, 2017) predicting unregulated stomatal aperture when the cost function is 0.

Notes S3 The leaf net carbon assimilation (A; mol CO_2 m^{-2} s^{-1}) in the Collatz et al (1992, 1991) model is calculated for C_3 and C_4 biochemical pathways as the co-limited rate of three potentially limiting gross carbon assimilation rates (W; mol CO_2 m^{-2} s^{-1}; Clark et al 2011). These rates are the Rubisco limited rate (W_c), light-limited rate (W_l) and transport limited rate (W_e) and are computed as described in equations S2.3 from Notes S2. The W_c and W_l for C_3 plants and the W_e for C_4 plants are solved in JULES using the c_i value produced by the Jacobs (1994) equation (equation 8 from the main text). We rearranged the equations from the Collatz (1991;1992) model in function of leaf stomatal conductance to CO_2 (g_s, mol m^{-2} s^{-1}), so we can use the g_s

produced by SOX (equation 4 from the main text) to compute *A*. This is done by substituting the c_i term in the equations S2.3 from Notes S2 by:

$$c_i = c_a - \frac{A}{g_s/P_a} \quad \text{(S3.1)}$$

The *A* term in equation S3.1 is equal to

$$A = W - R_d \quad \text{(S3.2)}$$

where R_d is the leaf dark respiration (mol CO_2 m^{-2} s^{-1}) calculated as a fraction of V_{cmax}:

$$R_d = f_d V_{cmax} \quad \text{(S3.3)}$$

The W_l and W_c for C_3 plants can be written generically as:

$$W = \frac{a(c_i - \Gamma)}{(c_i + b)} \quad \text{(S3.4)}$$

where *a* is equal to V_{cmax} for W_c and $\alpha(1\text{-}\omega)I_{PAR}$ for W_l, and *b* is equal to $K_c(1+O_a/K_o)$ for W_c and 2Γ for W_l. Replacing equation S3.1 into S3.4 we have:

$$A = \frac{a(c_i - \Gamma)}{(c_i + b)} - R_d \quad \text{(S3.5)}$$

We can then substitute equations S3.1 and S3.2 into equation S3.5:

$$A(c_i + b) = a(c_i - \Gamma) - R_d(c_i + b) \quad \text{(S3.6a)}$$

$$A\left(c_a - \frac{A}{g_s/P_a} + b\right) = a\left(c_a - \frac{A}{g_s/P_a} - \Gamma\right) - R_d\left(c_a - \frac{A}{g_s/P_a} + b\right) \quad \text{(S3.6b)}$$

Equation S3.6 can be rearranged into a quadratic:

$$\frac{-A^2}{g_s/P_a} + A(c_a + b) = \frac{A}{g_s/P_a}(R_d - a) + ac_a - a\Gamma - R_d c_a - bR_d \quad \text{(S3.7a)}$$

or

$$A^2 + A\beta + g_s/P_a\,\gamma = 0 \quad \text{(S3.7b)}$$

where

$$\beta = (R_d - a) - g_s/P_a\,(c_a + b) \quad \text{(S3.7c)}$$

$$\gamma = ac_a - a\Gamma - R_d c_a - bR_d \quad \text{(S3.7d)}$$

The limiting W_l and W_c rates for C_3 plants are the physically meaningful root of equation S3.7b, which can be obtained analytically with the equation:

$$A = \frac{-\beta}{2} - \sqrt{\left(\frac{\beta}{2}\right)^2 - \gamma} \quad \text{(S3.8a)}$$

and replacing equation S3.2 into S3.8a gives:

$$W = \left[\frac{-\beta}{2} - \sqrt{\left(\frac{\beta}{2}\right)^2 - \gamma}\right] + R_d \quad \text{(S3.8b)}$$

For C_4 plants there is a linear dependency between W_e and c_i in equation S2.3c (Notes S2), using the equation S3.1 and S3.2 we can derive the following expression for C_4 W_e:

$$A = kV_{cmax}\frac{\left(c_a - \frac{A}{g_s/P_a}\right)}{P_a} - R_d \quad \text{(S3.9)}$$

which can be rearranged into:

$$AP_a = kV_{cmax}c_a - \frac{kV_{cmax}A}{g_s/P_a} - R_dP_a \quad \text{(S3.10a)}$$

$$A = \frac{-g_s(R_dP_a - 2kV_{cmax}c_a)}{P_a(kV_{cmax}+g_s)} \quad \text{(S3.10b)}$$

$$W_e = \left[\frac{g_s(kV_{cmax}c_a - R_dP_a)}{P_a(kV_{cmax}+g_s)}\right] + R_d \quad \text{(S3.10c)}$$

Notes S4 The whole plant minimum hydraulic resistance (r_{pmin}) was calculated using the same approach from Eller *et al.* (2018) which followed Christoffersen *et al.* (2016) and Savage *et al.* (2010). The r_{pmin} was computed on a leaf area basis from the maximum petiole level hydraulic conductivity, $K_{\mathrm{pet,max}}$ (mol m^{-1} s^{-1} MPa^{-1}), the ratio between active xylem area and leaf area (i.e. Huber value, h_v; m^2 m^{-2}), maximum canopy height (h; m), and a unitless tapering factor (χ_{tap}) to account for changes in conduit diameter within trees:

$$r_{pmin} = \frac{h}{K_{pet,max}h_v}\chi_{tap} \quad (S4.1)$$

The $K_{pet,max}$ is calculated from maximum branch xylem conductivity ($K_{x,max}$; mol m^{-1} s^{-1} MPa^{-1}) following Christoffersen *et al.* (2016):

$$K_{pet,max} = K_{x,max}\left(\frac{r_{int,pet}}{r_{int,ref}}\right)^2 \quad (S4.2)$$

where $r_{int,pet}$ is the petiole conduit radius is assumed to be 10 µm and the $r_{int,ref}$ is the radius of conduits of the terminal branches set to 22 µm, to account for the conduit tapering from branch to petiole. We define the hydraulic tapering factor (χ_{tap}) as

$$\chi_{tap} = \frac{\chi_{tap:notap}(h)}{\chi_{tap:notap}(1)} \quad (S4.3)$$

where $\chi_{\mathrm{tap:notap}}(h)$ and $\chi_{\mathrm{tap:notap}}(1)$ are factors that represents the ratio of the theoretical whole tree conductance with taper ($K_{max,tap}$) to that without taper ($K_{max,notap}$) at height h and 1 m, respectively. These factors are calculated following the Savage *et al.* (2010) model as described by Christoffersen *et al.* (2016).

$$K_{max} = a\left(n_{ext}^{N/2}\right)^b \quad (S4.4)$$

where a and b are constants set to 7.2 x10^{-13} and 1.32, to calculate $K_{max,notap}$ and 6.6 x10^{-13} and 1.85 for $K_{max,tap}$ (Christoffersen *et al.*, 2016). The n_{ext} represents the branching patterns in the Savage *et al.* (2010), that is the number of daughter branches produced by each parent branch, which is assumed to be 2. The N is the total number of branching levels, calculated as a function of h:

$$N = \frac{3\ln\left[1 - \frac{h}{L_{pet}}(1 - n_{ext}^{1/3})\right]}{\ln(n_{ext})} - 1 \qquad (S4.5)$$

where L_{pet} is petiole length assumed to be 0.04 m.

References

De Angelis P, Scarascia-Mugnozza GE. **1998**. Long-term CO2-enrichment in a Mediterranean natural forest: An application of large open top chambers. *Chemosphere* **36**: 763–770.

Barton CVM, Duursma RA, Medlyn BE, Ellsworth DS, Eamus D, Tissue DT, Adams MA, Conroy J, Crous KY, Liberloo M, *et al.* 2012. Effects of elevated atmospheric [CO2] on instantaneous transpiration efficiency at leaf and canopy scales in *Eucalyptus saligna*. *Global Change Biology* **18**: 585–595.

Best MJ, Pryor M, Clark DB, Rooney GG, Essery R, Ménard CB, Edwards JM, Hendry MA, Porson A, Gedney N. 2011. The Joint UK Land Environment Simulator (JULES), model description–Part 1: energy and water fluxes. *Geoscientific Model Development* **4**: 677–699.

Buckley TN. **2017**. Modeling Stomatal Conductance. *Plant Physiology* **174**: 572–582.

Christoffersen BO, Gloor M, Fauset S, Fyllas NM, Galbraith DR, Baker TR, Kruijt B, Rowland L, Fisher RA, Binks OJ, *et al.* 2016. Linking hydraulic traits to tropical forest function in a size-structured and trait-driven model (TFS v.1-Hydro). *Geoscientific Model Development* **9**: 4227–4255.

Clark DB, Mercado LM, Sitch S, Jones CD, Gedney N, Best MJ, Pryor M, Rooney GG, Essery RLH, Blyth E, et al. 2011. The Joint UK Land Environment Simulator (JULES), Model description – Part 2: Carbon fluxes and vegetation. *Geoscientific Model Development* **4**: 641–688.

Collatz GJ, Ball JT, Grivet C, Berry JA. **1991**. Physiological and environmental regulation of stomatal conductance, photosynthesis and transpiration: a model that includes a laminar boundary layer. *Agricultural and Forest Meteorology* **54**: 107–136.

Collatz G, Ribas-Carbo M, Berry J. **1992**. Coupled Photosynthesis-Stomatal Conductance Model for Leaves of C 4 Plants. *Australian Journal of Plant Physiology* **19**: 519–538.

Eller CB, Lima AL, Oliveira RS. **2016**. Cloud forest trees with higher foliar water uptake capacity and anisohydric behavior are more vulnerable to drought and climate change. *The New phytologist* **211**: 489–501.

Eller CB, Rowland L, Oliveira RS, Bittencourt PRL, Barros FV, da Costa ACL, Meir P, Friend AD, Mencuccini M, Sitch S, *et al.* 2018. Modelling tropical forest responses to drought and El Niño with a stomatal optimization model based on xylem hydraulics. *Philosophical transactions of the Royal Society of London. Series B, Biological sciences* **373**: 20170315.

Gimeno TE, Crous KY, Cooke J, O'Grady AP, Ósvaldsson A, Medlyn BE, Ellsworth DS. **2016**. Conserved stomatal behaviour under elevated CO2 and varying water availability in a mature woodland. *Functional Ecology* **30**: 700–709.

Harper AB, Cox PM, Friedlingstein P, Wiltshire AJ, Jones CD, Sitch S, Mercado LM, Groenendijk M, Robertson E, Kattge J, et al. 2016. Improved representation of plant functional types and physiology in the Joint UK Land Environment Simulator (JULES v4.2) using plant trait information. *Geoscientific Model Development* **9**: 2415–2440.

Héroult A, Lin YS, Bourne A, Medlyn BE, Ellsworth DS. **2013**. Optimal stomatal conductance in relation to photosynthesis in climatically contrasting Eucalyptus species under drought. *Plant, Cell and Environment* **36**: 262–274.

Jacobs C. 1994. *Direct impact of atmospheric CO2 enrichment on regional transpiration*. PhD thesis, Wageningen Agricultural University, Wageningen, the Netherlands.

Limousin JM, Bickford CP, Dickman LT, Pangle RE, Hudson PJ, Boutz AL, Gehres N, Osuna JL, Pockman WT, Mcdowell NG. **2013**. Regulation and acclimation of leaf gas exchange in a piñon-juniper woodland exposed to three different precipitation regimes. *Plant, Cell and Environment* **36**: 1812–1825.

Mitchell PJ, Veneklaas E, Lambers H, Burgess SSO. **2009**. Partitioning of evapotranspiration in a semi-arid eucalypt woodland in south-western Australia. *Agricultural and Forest Meteorology* **149**: 25–37.

Martin-St. Paul NK, Limousin JM, Rodrígesuez-Calcerrada J, Ruffault J, Rambal S, Letts MG, Misson L. **2012**. Photosynthetic sensitivity to drought varies among populations of *Quercus ilex* along a rainfall gradient. *Functional Plant Biology* **39**: 25–37.

Nash JE, Sutcliffe JV. 1970. River flow forecasting through conceptual models part I — A discussion of principles. *Journal of Hydrology* **10**: 282–290.

Rowland L, Lobo-do-Vale RL, Christoffersen BO, Melém EA, Kruijt B, Vasconcelos SS, Domingues T, Binks OJ, Oliveira AAR, Metcalfe D, *et al.* 2015. After more than a decade of soil moisture deficit, tropical rainforest trees maintain photosynthetic capacity, despite increased leaf respiration. *Global Change Biology* **21**: 4662–4672.

Savage VM, Bentley LP, Enquist BJ, Sperry JS, Smith DD, Reich PB, von Allmen EI. **2010**. Hydraulic trade-offs and space filling enable better predictions of vascular structure and function in plants. *Proceedings of the National Academy of Sciences of the United States of America* **107**: 22722–7.

Sperry JS, Love DM. **2015**. What plant hydraulics can tell us about responses to climate-change droughts. *New Phytologist* **207**: 14–27.

Sperry JS, Venturas MD, Anderegg WRL, Mencuccini M, Mackay DS, Wang Y, Love DM. **2017**. Predicting stomatal responses to the environment from the optimization of photosynthetic gain and hydraulic cost. *Plant Cell and Environment* **40**: 816–830.

Teodoro GS. **2014**. *Extreme drought effects on the phenology, growth and ecophysiology performance of campos rupestres species*. PhD Thesis, University of Campinas, Campinas, Brazil.

Thomas DS, Eamus D. **2002**. Seasonal patterns of xylem sap pH, xylem abscisic acid concentration, leaf water potential and stomatal conductance of six evergreen and deciduous Australian savanna tree species. *Australian Journal of Botany* **50**: 229–236.

Twine TE, Kustas WP, Norman JM, Cook DR, Houser P, Meyers TP, Prueger JH, Starks PJ, Wesely ML. 2000. Correcting eddy-covariance flux underestimates over a grassland. *Agricultural and Forest Meteorology* **103**: 279-300.

Wolf A, Anderegg WRL, Pacala SW. **2016**. Optimal stomatal behavior with competition for water and risk of hydraulic impairment. *Proceedings of the National Academy of Sciences* **113**: E7222–E7230.

Zeppel M, MacInnis-Ng C, Palmer A, Taylor D, Whitley R, Fuentes S, Yunusa I, Williams M, Eamus D. **2008**. An analysis of the sensitivity of sap flux to soil and plant variables assessed for an Australian woodland using a soil-plant-atmosphere model. *Functional Plant Biology* **35**: 509–520.
